# Widespread higher fractional anisotropy associates to better cognitive functions in individuals at ultra‐high risk for psychosis

**DOI:** 10.1002/hbm.24765

**Published:** 2019-08-20

**Authors:** Tina D. Kristensen, René C. W. Mandl, Jayachandra M. Raghava, Kasper Jessen, Jens Richardt M. Jepsen, Birgitte Fagerlund, Louise B. Glenthøj, Christina Wenneberg, Kristine Krakauer, Christos Pantelis, Merete Nordentoft, Birte Y. Glenthøj, Bjørn H. Ebdrup

**Affiliations:** ^1^ Copenhagen Research Center for Mental Health, CORE, Mental Health Centre Copenhagen University of Copenhagen Hellerup Denmark; ^2^ Center for Clinical Intervention and Neuropsychiatric Schizophrenia Research, CINS, and Center for Neuropsychiatric Schizophrenia Research, CNSR, Mental Health Centre Glostrup University of Copenhagen Glostrup Denmark; ^3^ University Medical Center Utrecht Brain Center Utrecht the Netherlands; ^4^ Functional Imaging Unit, Department of Clinical Physiology, Nuclear Medicine and PET University of Copenhagen Glostrup Denmark; ^5^ Child and Adolescent Mental Health Centre, Mental Health Services, Capital Region of Denmark University of Copenhagen Hellerup Denmark; ^6^ Department of Psychology, Faculty of Social Sciences University of Copenhagen Copenhagen Denmark; ^7^ Melbourne Neuropsychiatry Center, MNC The University of Melbourne Melbourne Australia; ^8^ Department of Clinical Medicine, Faculty of Health and Medical Sciences University of Copenhagen Copenhagen Denmark

**Keywords:** cognition, diffusion tensor imaging, partial least squares correlation analysis, ultra‐high risk for psychosis, white matter

## Abstract

In schizophrenia patients, cognitive functions appear linked to widespread alterations in cerebral white matter microstructure. Here we examine patterns of associations between regional white matter and cognitive functions in individuals at ultra‐high risk for psychosis. One hundred and sixteen individuals at ultra‐high risk for psychosis and 49 matched healthy controls underwent 3 T magnetic resonance diffusion‐weighted imaging and cognitive assessments. Group differences on fractional anisotropy were tested using tract‐based spatial statistics. Group differences in cognitive functions, voxel‐wise as well as regional fractional anisotropy were tested using univariate general linear modeling. Multivariate partial least squares correlation analyses tested for associations between patterns of regional fractional anisotropy and cognitive functions. Univariate analyses revealed significant impairments on cognitive functions and lower fractional anisotropy in superior longitudinal fasciculus and cingulate gyrus in individuals at ultra‐high risk for psychosis. Partial least squares correlation analysis revealed different associations between patterns of regional fractional anisotropy and cognitive functions in individuals at ultra‐high risk for psychosis compared to healthy controls. Widespread higher fractional anisotropy was associated with better cognitive functioning for individuals at ultra‐high risk for psychosis, but not for the healthy controls. Furthermore, patterns of cognitive functions were associated with an interaction‐effect on regional fractional anisotropy in fornix, medial lemniscus, uncinate fasciculus, and superior cerebellar peduncle. Aberrant associations between patterns of cognitive functions to white matter may be explained by dysmyelination.

## 1 INTRODUCTION

The ultra‐high risk (UHR) criteria (Yung, Yuen, Phillips, Francey, & McGorry, 2003) are commonly used to identify individuals at heightened risk for developing a psychosis. Research on UHR‐individuals is critical for early intervention, considering the detrimental individual and societal effects of psychosis (Bora, Yucel, & Pantelis, 2009; Pantelis et al., 2005). Regardless of transition to psychosis, UHR‐individuals are characterized by persistent impairments in cognition and functioning (Nelson et al., 2010; Simon et al., 2013).

UHR‐individuals display impairments across multiple cognitive domains (Bora & Murray, 2014; Glenthøj et al., 2018), with an overall small to medium effect size compared to healthy controls (HCs; Fusar‐Poli et al., 2012). Importantly, cognitive deficits are predictive of functional outcome (Niendam, Jalbrzikowski, & Bearden, 2009), and impairments in executive functions, such as verbal memory, fluency, and working memory may be predictive of transition to psychosis (Kim et al., 2011).

White matter (WM) consists of fiber bundles of neuronal axons running in parallel, providing efficient information transfer between cortical regions (Peer, Nitzan, Bick, Levin, & Arzy, 2017). Consequently, impairments of WM may lead to a disturbed communication between regions, which may impair cognitive functioning (Borghesani et al., 2013). Since higher‐order cognitive functions require communication between distributed brain‐regions (Bressler & Menon, 2010; Filley & Fields, 2016), linking WM and cognition may provide valuable information on the functional implications of altered WM‐microstructure affecting the course of illness (Friston, 2011).

WM‐abnormalities have been observed in a broad spectrum of psychiatric disorders, including UHR‐individuals (Bora et al., 2011; Fields, 2008; Jenkins et al., 2016). Associations between WM and cognition have been established through neuroimaging studies in diverse populations (Kochunov et al., 2017; Magioncalda, Martino, Ely, Inglese, & Stern, 2016; Schaeffer et al., 2015). However, these studies are characterized by modest sample‐sizes and the results are equivocal. Here we examine, if there is a differential pattern of associations between WM‐microstructure and cognitive functions in UHR‐individuals compared to HC.

Diffusion‐weighted imaging (DWI) is a noninvasive magnetic resonance imaging (MRI) technique and is the method of choice to study various aspects of WM‐microstructure in vivo (Concha, 2014). With DWI, we can measure the size and shape of the diffusion profile of water molecules in brain tissue. In WM, the water molecules diffuse more easily along the WM‐fiber bundles and tend to be more hindered in the perpendicular directions (Johansen‐Berg & Behrens, 2009). Currently, the most widely applied DWI‐derived measure is fractional anisotropy (FA; Beaulieu, 2002; Le Bihan & Iima, 2015). FA serves as a sensitive, but nonspecific (Alexander, Lee, Lazar, & Field, 2007) index of WM‐organization. The interpretation of underlying biological processes is aided by applying other DWI‐derived indices, such as axial diffusivity (AD), radial diffusivity (RD), and mean diffusivity (MD). Specific combinations of these indices have been linked to respectively dysmyelination, axonal damage, and inflammation (Alexander et al., 2007, 2011; Table S5). However, one should be cautious with the interpretation, since these indices may be influenced by multiple factors such as image‐noise and crossing fibers.

Partial least square correlation (PLS‐C) is a multivariate analysis method (Krishnan, Williams, McIntosh, & Abdi, 2011), offering advantageous features in the modeling of complex associations, for example, interactions and correlations between multiple neuropsychological and neuroimaging indices (Sarstedt, Hair, Ringle, Thiele, & Gudergan, 2016). Thus, PLS‐C provides a mean for investigating the functional implications of altered WM‐microstructure. Moreover, the effect‐sizes of the WM‐alterations in UHR‐individuals are expected to be small, and therefore univariate statistical analysis is vulnerable to Type‐2 errors, when a correction for multiple comparisons is applied. When applying PLS‐C, we benefit from the larger sensitivity to obtain additional and complementary information on the complex relations between the expected subtle cerebral changes and widespread cognitive impairments in UHR‐individuals. Recently, we applied a similar PLS‐C approach in independent studies of UHR‐individuals (Krakauer et al., 2017) and first‐episode psychotic patients [Jessen et al., 2018].

In the current study, we expected impairments in various cognitive functions, and widespread subtle WM‐alterations characterized by lower FA in UHR compared to HC.

## 2 METHODS

The study was conducted in accordance with the declaration of Helsinki. The study protocol was approved by the Committee on Health Research Ethics of the Capital Region Denmark (H‐6‐2013‐015), and the Danish Data Protection Agency (2007‐58‐0015, I‐Suite no. 02670). All participants provided informed oral and written consent prior to inclusion.

### 2.1 Participants

Participants were recruited as part of a randomized clinical trial examining the effect of cognitive remediation in UHR‐individuals at the Mental Health Centre Copenhagen, Denmark (the FOCUS‐trial; Glenthøj et al., 2015). A total of 116 patients were recruited from the adult psychiatric in‐ and outpatient facilities in the catchment area of Copenhagen, between April 2014 and December 2017. We have previously presented cognitive data on subsamples of UHR‐individuals compared to HC (Glenthøj, Fagerlund, et al., 2016; Glenthøj, Jepsen, et al., 2016; Glenthøj et al., 2018). The current study includes baseline data on MR‐DWI and cognition. UHR‐individuals were help‐seeking, aged 18–40 years, and fulfilled one or more of the UHR‐criteria as assessed by the Comprehensive Assessment of At Risk Mental State (CAARMS; Yung et al., 2005): attenuated psychotic symptoms, brief limited psychotic episodes, or state‐and‐trait vulnerability (a first‐degree relative with psychotic disorder, or a diagnose of schizotypal personality disorder). Exclusion criteria were: psychiatric symptoms only co‐occurring with acute intoxication, organic brain disease, a diagnosis of a developmental disorder, current treatment with methylphenidate, or MRI scans with overt pathology as evaluated by a trained neuroradiologist. HC were concurrently included and matched 1:2 to the UHR‐individuals on age, gender, ethnicity, and parental socioeconomic status (SES). HC were recruited through internet and community‐based advertising, and had no current or previous psychiatric diagnoses, substance abuse or dependency, and no first‐degree relative with a psychotic disorder.

### 2.2 Assessments

#### 2.2.1 Clinical measures

The CAARMS interview and The Structured Clinical Interview for DSM‐IV Axis I Disorders (SCID‐I) and part of the Structured Clinical Interview for DSM‐IV Axis II Disorders (SCID‐II; First & Gibbon, 2004; Spitzer, Williams, Gibbon, & First, 1990) were used to diagnostically assess all participants. Trained assessors were experienced psychologists and medical doctors.

#### 2.2.2 Cognitive measures

Premorbid IQ was estimated using the Danish adaptation of the National Adult Reading Test (DART; Bright, Jaldow, & Kopelman, 2002). The third version of the Danish Weschler Adult Intelligence Scale (WAIS‐III; Wechsler, 1997) provided estimates of: (a) current verbal IQ using the Similarities subtest, and (b) current performance IQ using the Block Design subtest. Cognitive functions were assessed using selected tests from the Brief Assessment of Cognition in Schizophrenia battery (Keefe et al., 2008): list‐Learning, digit sequencing, fluency, and symbol coding; as well as tests from the Cambridge Neuropsychological Test Automated Battery (CANTAB; Sahakian & Owen, 1992): spatial working memory (SWM), stockings of Cambridge (SOC), intraextradimensional set shifting test (IED), paired associate learning (PAL), reaction time index (RTI), and rapid visual information processing (RVP). For a detailed overview on cognitive domains and tests, see Table S1.

#### 2.2.3 Image acquisition and processing

MRI scans were acquired on a 3 T scanner (Philips Healthcare, Best, the Netherlands). Diffusion‐weighted images (DWIs) were acquired using single shot spin‐echo echoplanar imaging (EPI) sequence with 30 noncollinear diffusion‐weighted (*b* = 1,000 s/mm^2^). Two DWI scans were acquired, and the latter was acquired in an opposite phase encoding direction, enabling correction for susceptibility distortions (Andersson, Skare, & Ashburner, 2003; for details on image acquisition and processing, see Text S1).

Tools from the FSL software library v5.0.10 (Jenkinson, Beckmann, Behrens, Woolrich, & Smith, 2012) and MRtrix3 (http://www.mrtrix.org) were used for image processing. Motion parameters were extracted to correct for head motion. FA maps were calculated and tract‐based spatial statistics (TBSS; Smith et al., 2006) was used to create skeleton maps using a threshold of 0.2. For exploratory analyses, skeleton maps were similarly created for AD, RD, and MD. Using the JHU DTI‐based WM atlas labels (Mori & Zijl, 2007), we extracted the mean FA, AD, RD, and MD values in 48 WM label regions of interest (ROIs) from skeletonized data (Figure S1). MRI quality metrics were assessed by visual inspection and calculated from each subject using a quality assessment method described in Roalf et al. (2016) (Table S2).

### 2.3 Statistical analysis

Demographic and clinical data were analyzed with univariate tests using IBM SPSS Statistics for Windows, Version 25.0, Armonk, NY.

To test potential group differences in cognitive functions and WM, we first applied univariate GLM‐analyses on cognitive data comparing UHR‐individuals to HC. Next, to obtain a more complete picture, we also examined WM alterations using two complimentary univariate methods: a standard whole brain voxel‐wise analysis and ROI‐analysis of the skeletonized FA data. Finally, as our main analysis we applied multivariate PLS‐C to explore associations between patterns of cognitive functions and regional FA for UHR‐individuals compared to HC.

### 2.4 Univariate analyses

#### 2.4.1 Demographic and clinical data

Distribution of continuous data was tested for normality, and group differences were tested using GLM co‐varied for age and gender. Group differences in ordinal data regarding tobacco smoking, alcohol, and drug use were tested using the Mann–Whitney *U* test or Fisher's exact test. Nominal data were tested using Pearson's *χ* ^2^ test.

#### 2.4.2 Cognitive data

Distribution of data from 16 cognitive tests was tested for normality, and in case of inhomogeneity or nonnormality, the cognitive data were transformed (Templeton & Templeton, 2011). Outliers were identified using the interquartile range (IQR)‐method (Sokal & Rohlf, 1994). We tested group differences using GLM and co‐varied for age and gender. Effect size was calculated with Hedges' *d*. Results were corrected for multiple comparisons using Bonferroni correction (Jean Dunn, 1961), with significance threshold set at *p* = .003 (0.05/16).

#### 2.4.3 Magnetic resonance imaging data

Whole brain voxel‐wise group differences were analyzed on the skeletonized FA data using *randomize* (version 2.1) with 5,000 permutations (Winkler, Ridgway, Webster, Smith, & Nichols, 2014), co‐varied for age, gender, parental SES, tobacco smoking, alcohol consumption, and relative and absolute movement in scanner. Family‐wise error correction (Bullmore et al., 1999) with a threshold of *p* < .05 was used to correct for multiple comparisons, and threshold‐free cluster enhancement (Smith & Nichols, 2009) was applied. The anatomical locations of significant clusters were identified in MNI space using the JHU WM tractography atlas (Mori & Zijl, 2007; Figure S1). Group differences on 48 regional FA were tested using GLM and co‐varied for age, gender, parental SES, tobacco smoking, alcohol consumption, and relative and absolute head motion in scanner. Results was corrected for multiple comparisons using Bonferroni correction (Jean Dunn, 1961) with significance threshold set at *p* = .001 (0.05/48).

### 2.5 Multivariate PLS‐C analyses

**Figure 1:**
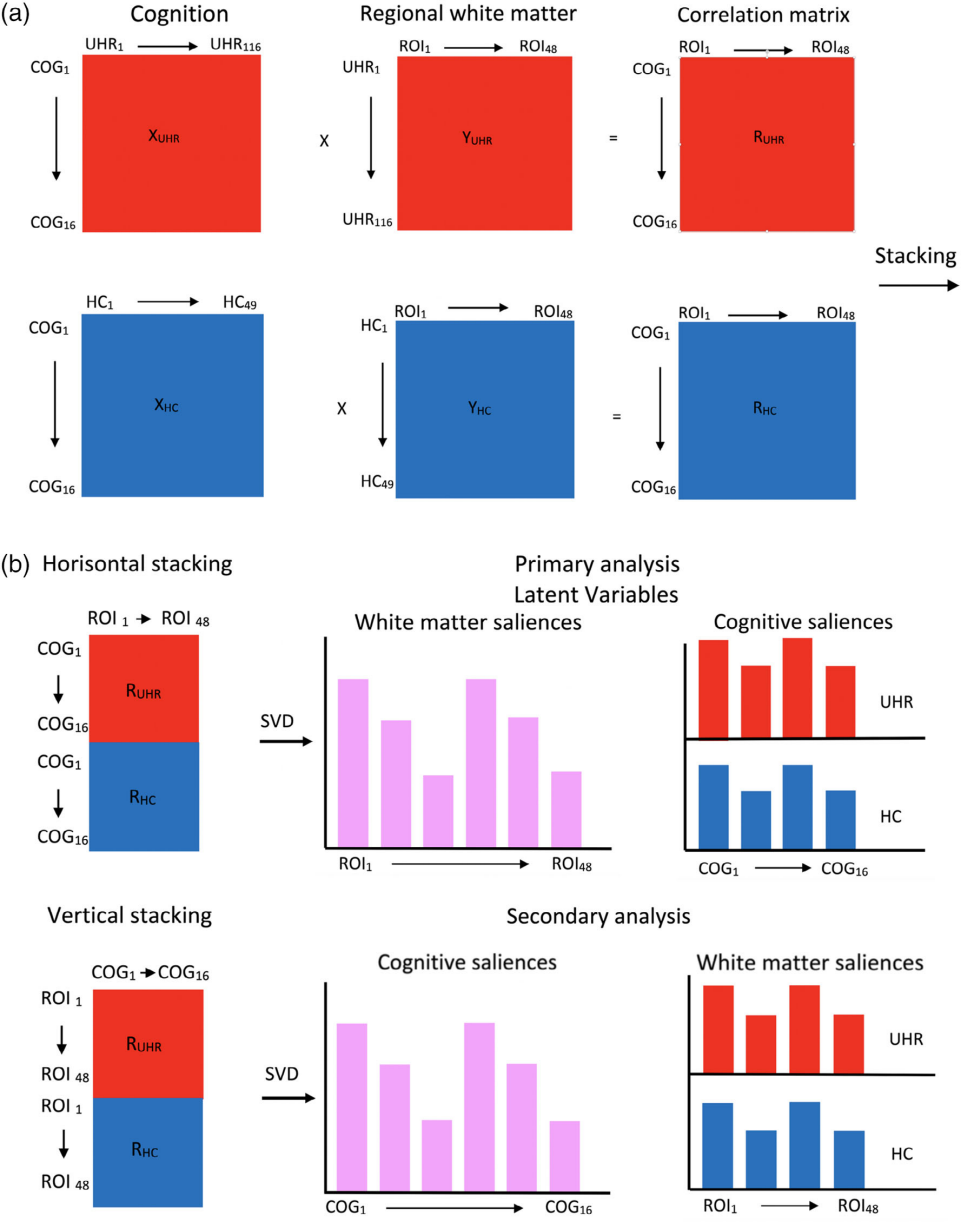
PLS‐C for between‐group analysis. We included 16 cognitive functions and mean FA values of 48 WM‐regions. First, a correlation matrix (R) was computed between cognitive functions and FA data (Figure 1a. These correlation matrixes were calculated for each group (UHR and HC) and stacked horizontal and vertically (Figure 1b). When stacked horizontally, we examined between‐group differences in patterns of cognitive functions, associated with a shared pattern of regional FA (“primary analysis”). Reversely when stacked vertically, we examined between‐group differences in patterns of regional FA, associated with a shared pattern of cognitive functions (“secondary analysis”). In next step, the correlation matrixes were decomposed using singular value decomposition (SVD) to obtain latent variables (LVs). LVs express the maximum covariance between patterns of cognitive functions and group‐specific regional FA. All LVs combined completely describe the covariance matrix (100%). However, in our study we were interested in LVs, that captured the part of the covariance matrix describing the differences between UHR‐individuals and healthy controls. If this difference is evident enough, then the LV is significant (UHR‐individuals show a different association between cognitive functions and regional FA compared to healthy controls). The contribution of each of the cognitive functions and ROIs to the latent variable can be determined by projecting a significant LV back to the original variables. These projections are known as saliences. We used the two‐group PLS‐C analysis (see Jessen et al. (2018)) in an extended version to analyze the interaction‐effects in the first step of the analysis. Detailed explanation of the extra shuffling across groups during the permutations testing for interaction is illustrated in Figure S2. The second step of analysis, involving unpaired shuffling in the permutations testing, can be performed to test correlation‐effects. Abbreviations: COG, cognitive outcomes; FA, fractional anisotropy; FSL, FMRIB Software Library; HCs, Healthy controls; JHU, John Hopkins University; R, cross‐block correlation matrix; ROI, region of interest; SV, singular value; SVD, singular value decomposition; UHR, ultra‐high risk of psychosis; X, matrix of ROI FA values; Y, matrix of cognitive outcomes [Color figure can be viewed at http://wileyonlinelibrary.com]

The main PLS‐C analysis (Abdi & Williams, 2013; McIntosh & Lobaugh, 2004) was performed using the MATLAB software (version 2017b). We included 16 cognitive functions and mean FA values of 48 WM‐regions, co‐varied for age, gender, parental SES, tobacco smoking, alcohol consumption, and relative and absolute movement in scanner. We used the two group PLS‐C analysis (Jessen et al., 2018) to analyze associations between regional WM and cognitive function. In short, here PLS‐C is used to identify latent variables (LVs), which express maximum covariance between: (a) patterns of regional FA associated with group‐specific cognitive functions and conversely (b) patterns of cognitive functions associated with group‐specific regional FA (see detailed illustration in Figure 1). Both the significance level of the omnibus test (Reisfeld & Mayeno, 2013) and of the individual LVs were assessed using permutation testing (100.000 permutations) to obtain a *p*‐value based on nonrotated sampling distribution of singular values (Kovacevic, Abdi, Beaton, & McIntosh, 2013). For the omnibus test, the Inertia index that was calculated as the sum of all singular values of all the LVs identified by PLS‐C, was used for permutations testing (Abdi & Williams, 2013). LVs with a *p*‐value below .05 were considered significant. Only LVs with a cross‐block covariance larger than 5 % were reported (Grigg & Grady, 2010). The reliability of saliences was assessed using bootstrapping (100.000 bootstraps with procrustean rotation) to obtain 95% confidence intervals. Confidence intervals of the saliences that did not cross zero were considered reliable (Krishnan et al., 2011).

### 2.6 Exploratory analyses

To determine whether potential group differences on the cognitive functions could be regarded as an effect of between‐group difference in intelligence, we tested group differences on cognitive functions with additional covariation for premorbid IQ.

As sensitivity analysis, we examined the potential effect of medication (Ebdrup, Raghava, Nielsen, Rostrup, & Glenthøj, 2015), substance abuse and dependency on group differences on regional FA by performing three exploratory univariate GLM‐analyses. Firstly, we compared HC with antipsychotic‐naïve UHR‐individuals. Secondly, we compared HC with psychotropic‐medication‐naïve UHR‐individuals (no lifetime exposure to antipsychotics, antidepressants, mood‐stabilizers, or benzodiazepines), and finally we compared HC with psychotropic‐medication‐naïve combined with substance abuse and dependency‐naïve UHR‐individuals. Furthermore, UHR‐individuals with substance abuse‐ and dependency were examined regarding outlier‐values on mean global FA.

For regions demonstrating interaction‐effect on FA, we performed exploratory analyses of covariance (ANCOVA) by correlating cognition scores with FA, AD, RD, and MD. Cognition scores were calculated using cognition saliences from the specific LVs (Krishnan et al., 2011).

## 3 RESULTS

### 3.1 Univariate analyses

#### 3.1.1 Demographic and clinical characteristics

Demographic and clinical data are presented in Table 1. The UHR‐individuals and HC did not differ significantly in age, gender, parental SES, ethnicity, body‐mass index, handedness, or recreational use of cannabis. HC had a significantly larger recreational alcohol consumption, and UHR‐individuals had more tobacco use.

**Table 1:** Demographic and clinical characteristics

Variable	UHR‐individuals (*N* = 116)	Healthy controls (*N* = 49)	*p*‐value
	Percent/mean (*SD*)	Percent/mean (*SD*)	
*Age* mean (SD)	23.8 (4.2)	24.4 (3.4)	.37
*Gender* (percent)			.87
Male/female	47.4/52.6	44.9/55.1	
*Parental SES* (percent)			.35
Low	10.3	4.1	
Medium	37.1	34.7	
High	52.6	61.2	
*Ethnicity* (percent)			.35
High‐income countries	91.0	95.9	
Low‐income countries	9.0	4.1	
*BMI* mean (SD)	23.4 (4.7)	23.3 (3.2)	.87
*Handedness* (percent)			.50
Right	86.2	87.8	
Left	13.8	12.2	
*Function*			
SOFAS mean (SD)	55.1 (11.0)	89.0 (4.9)	<.01
*Alcohol consumption (last year)* (percent)			.02
Daily	1.8	4.1	
Weekly	30.6	44.9	
Monthly	38.7	46.9	
Once/twice	15.3	4.1	
Never	13.5	0.0	
*Tobacco smoking (last year)* (percent)			<.01
Daily	42.9	2.0	
Weekly	5.4	8.2	
Monthly	4.5	6.1	
Once/twice	4.5	2.0	
Never	42.9	81.6	
*Cannabis smoking (last year)* (percent)			.47
Daily	2.8	0.0	
Weekly	4.6	4.3	
Monthly	6.4	4.3	
Once/twice	15.6	26.1	
Never	70.6	65.2	
*Medication* (percent)			
Antipsychotic‐naive	57.8	100.0	
All medication naive	26.7	100.0	
Current antipsychotic	32.8	0.0	
Current antidepressant	27.6	0.0	
Current mood‐stabilizers	5.2	0.0	
Current benzodiazepines	7.8	0.0	
Diagnose of lifetime abuse	7.8	0.0	
Diagnose of current^a^ abuse	0.9	0.0	
Diagnose of lifetime dependency	9.5	0.0	
Diagnose of current^a^ dependency	0.9	0.0	‐
Medication naive and no substance abuse or dependency	24.1	100.0	
*CAARMS subgroups* (percent)			
APS	98.2	0.0	
BLIPS	2.6	0.0	
TS vulnerability	23.7	0.0	
*Diagnoses* (percent)			
Affective disorder	54.3	0.0	
Anxiety disorder	46.6	0.0	
Personality disorder	34.5	0.0	
Other diagnoses	18.1	0.0	
≥3 diagnoses	40.0	0.0	

*Note*: Table 1 shows the demographic and clinical characteristics for UHR and HC.

Abbreviations: APS, attenuated psychotic symptoms; BLIPS, brief limited intermittent psychotic symptoms; BMI, body‐mass index; CAARMS, comprehensive assessment of at‐risk mental state; SD, standard deviation; SES, socioeconomic status; SOFAS, social and occupational function assessment scale; TS, trait and state; UHR, ultra‐high risk.

a Current = the last month.

#### 3.1.2 Cognition

Cognitive data are presented in Table 2. UHR‐individuals performed significantly lower than HC on 14 out of 16 measures. Reaction time, visual learning and memory, and planning latency did not differ between groups. With Bonferroni correction, between‐group effect remained significant on 9 out of 16 measures, covering verbal learning, verbal fluency and working memory, processing speed, spatial working memory, planning abilities, latency in cognitive flexibility, and sustained attention. Exploratory analyses covarying for premorbid IQ on the cognitive functions further removed significant between‐group effects on one subtest from CANTAB (spatial working memory).

**Table 2:** Cognitive functions

	UHR‐individuals (*N* = 116)	Healthy controls (*N* = 49)	Significance
	Mean (*SD*)	Mean (*SD*)	Effect size
Cognitive measure	[95% CI]	[95% CI]	(Hedges' *g*)
*DART*	21.72 (7.13)	24.49 (7.51)	*p* = .04
	[7.46–35.98]	[9.47–39.51]	*g* = 0.38
*WAIS*			
• Verbal IQ	24.03 (4.86)	25.76 (3.62)	*p* = .04
(Similarities)	[14.31–33.75]	[18.52–33.00]	*g* = 0.38
• Performance IQ	53.67 (10.38)	58.29 (8.95)	*p* = .01^b^
(Block design)	[32.91–74.43]	[49.34–76.19]	*g* = 0.46
*BACS*			
• List‐learning	50.97 (8.44)	59.84 (5.63)	*p* < .01^b^
	[34.09–67.85]	[48.58–71.10]	*g* = 1.15
• Digit sequencing	20.86 (3.83)	23.16 (2.97)	*p* < .01^b^
	[13.20–28.52]	[17.22–29.10]	*g* = 0.64
• Fluency	58.08 (13.79)	69.94 (11.99)	*p* < .01^b^
	[30.50–85.66]	[45.96–93.92]	*g* = 0.89
• Symbol coding	58.20 (11.49)	69.41 (13.60)	*p* < .01^b^
	[35.22–81.18]	[42.21–96.61]	*g* = 0.92
*CANTAB*			
• SWM strategy^a^	27.07 (6.02)	25.08 (5.19)	*p* = .05
	[15.03–39.11]	[14.70–35.46]	*g* = 0.34
• SWM total errors^a^	10.87 (10.87)	7.10 (8.65)	*p* = .03^b^
	[−10.87–32.61]	[−10.2–24.40]	*g* = 0.37
• SOC problems solved in minimum moves	9.91 (1.76)	10.76 (1.38)	*p* < .01^b^
	[6.39–13.43]	[8.00–13.52]	*g* = 0.51
• SOC mean initial thinking time five moves	9999.40 (5958.93)	11416.91 (7717.29)	*p* = .22
	[−1919.46–21917.26]	[−3717.67–26851.49]	*g* = 0.22
• IED total errors adj.^a^	19.53 (17.10)	14.39 (14.33)	*p* = .05
	[−14.67–53.73]	[−14.27–43.05]	*g* = 0.32
• IED total latency^a^	150885.41 (42395.65)	133134.39 (36869.15)	*p* = .01^b^
	[66094.11–235676.71]	[59396.09–206872.69]	*g* = 0.44
• PAL total trials adj.^a^	10.28 (2.83)	9.53 (1.58)	*p* = .07
	[4.62–15.94]	[6.37–12.69]	*g* = 0.30
• RTI mean simple reaction time^a^	303.24 (54.85)	292.57 (32.00)	*p* = .20
	[193.54–412.94]	[228.57–356.57]	*g* = 0.22
• RVP A'	0.8967 (0.0545)	0.9396 (0.0450)	*p* < .01^b^
	[0.7877–1.0057]	[0.8496–1.0296]	*g* = 0.83

*Note*: Table 2 shows the results on cognitive functions for UHR‐individuals and HCs.

Abbreviations: Adj., adjusted; BACS, brief assessment of cognition in schizophrenia; CANTAB, Cambridge neuropsychological test automated battery; CI, confidence interval; DART, Danish adult reading list; GLM, general linear modeling; IED, intra‐extra dimensional shift; IQ, intelligence quotient; PAL, paired associated learning; RTI, reaction time index; RVP, rapid visual processing; SD, standard deviation; SOC, stockings of Cambridge; SWM, spatial visual memory; UHR, ultra‐high risk; WAIS, Wechsler's adult intelligence scale.

a A lower score is better. These scores were reversed for the PLS‐C analysis.

b Between‐group effect remained significant after Bonferroni correction.

#### 3.1.3 Whole brain voxel‐wise WM

**Figure 2:**
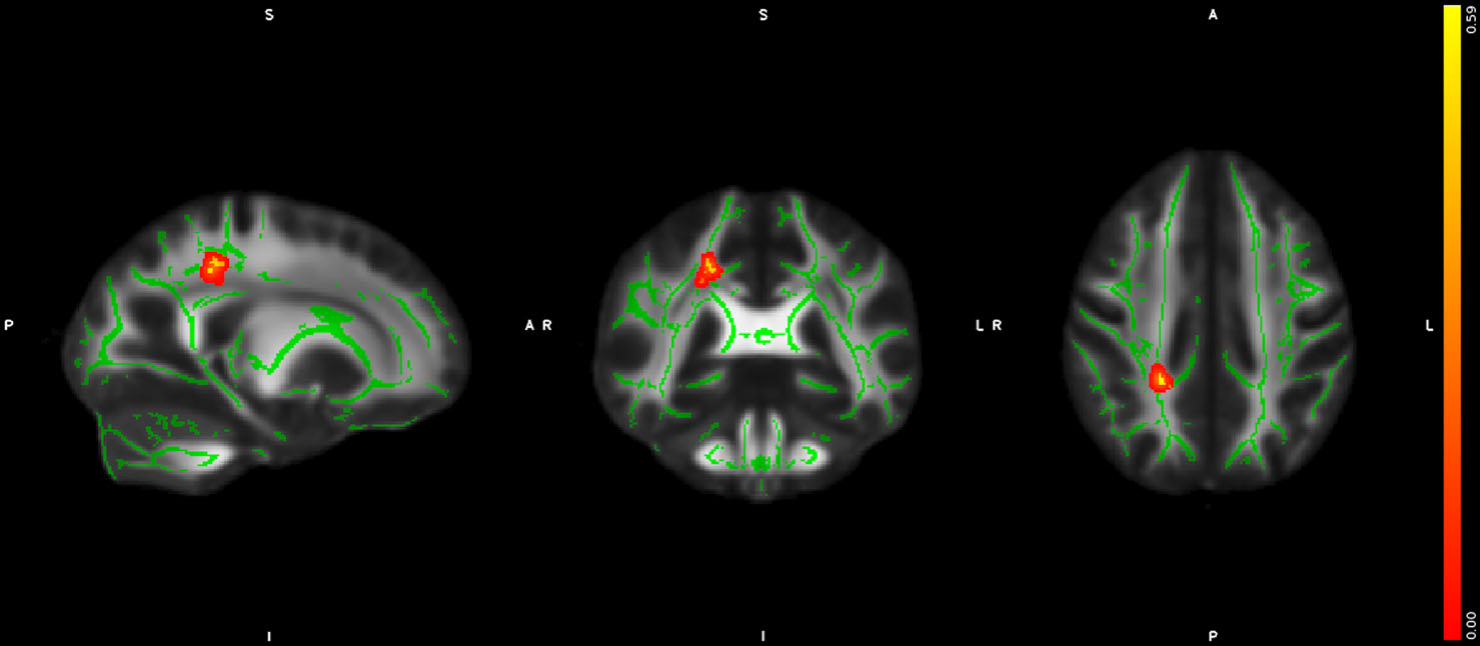
Voxel‐based between‐group difference on FA. The results from the univariate TBSS GLM‐analyses, revealing significant lower FA in UHR‐individuals compared to HC in the right superior longitudinal fasciculus and right cingulate gyrus. The cluster is enhanced for visualization using TBSS_fill and highlighted in red‐yellow colors and projected on the mean study‐specific FA‐skeleton template (green color). Abbreviations: FA, fractional anisotropy; GLM, general linear modeling; HCs, healthy controls; TBSS, tract‐based spatial statistics; UHR, ultra‐high risk of psychosis; WM, white matter [Color figure can be viewed at http://wileyonlinelibrary.com]

The voxel‐based GLM‐analyses on TBSS data revealed significant (*p* < .05, corrected), but focal WM‐abnormalities (lower FA) in UHR‐individuals compared to HC (Figure 2). The size of the cluster was 30 voxels, and the center of the mass was located at coordinates (21.5; −40.5; 40.9), predominantly linked to the right superior longitudinal fasciculus (SLF), expanding into cingulate gyrus white matter (CG).

#### 3.1.4 Regions of interest WM

Univariate GLM‐analysis revealed significant between‐group differences in 4 out of 48 ROIs. UHR‐individuals had significantly lower FA compared to HC in the right anterior corona radiata, right fornix (cres) stria terminalis, right SLF, and left tapetum (Table S3). The posthoc analyses for group differences on the ROIs remained significant in three out of four ROIs (right anterior corona radiata, right fornix (cres) stria terminalis, and right SLF), when comparing antipsychotic‐naïve UHR‐individuals with HC, but not when comparing psychotropic‐medication‐naïve UHR‐individuals or combined psychotropic‐medication, and substance abuse and dependency‐naïve UHR‐individuals with HC (Table S4). Effect‐sizes were medium, and significance levels did not survive Bonferroni correction (significance threshold set at *p* = .001).

### 3.2 Multivariate partial least squares correlation

The main between‐group PLS‐C interaction analysis revealed only trend‐level significant interaction in the associations between patterns of regional FA and cognitive functions in UHR‐individuals compared to HC (omnibus test *p* = .078). However, next step PLS‐C correlation analysis identified differential associations between a pattern of regional FA and cognitive functions in UHR‐individuals compared to HC (omnibus test *p* = .0085). Two significant latent variables (LV) were identified: LV1 explained 42% (*p* = .048) and LV6 explained 5% of the covariance (*p* = .0026). Since LV1 explained more than 5% of the covariance, only LV1 is further analyzed here.

**Figure 3:**
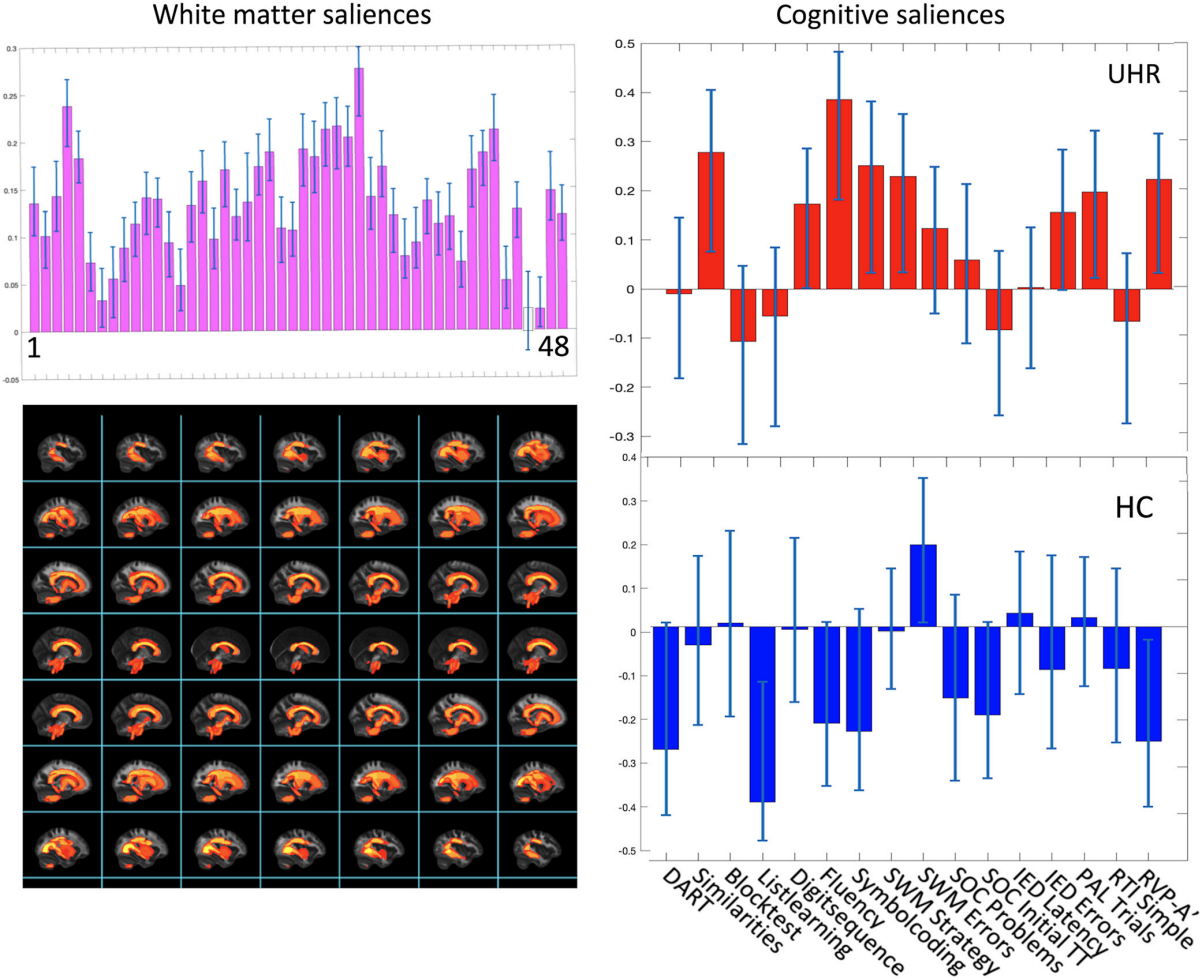
Primary PLS‐C analysis. The results from the primary PLS‐C analysis, illustrating LV1. Left column displays the White matter FA‐saliences of the 48 ROIs in purple bars. Bars colored gray does not contribute reliably to the positive correlation between white matter FA and cognitive functions. Confidence intervals are marked with light‐blue lines in each bar. Below, the regions with significant interaction‐effect is projected on a standard brain derived from JHU WM‐atlas. In the right column, the saliences of the 16 cognitive functions associated with the pattern of regional WM FA are displayed and stacked for each group separately, highlighted in red for UHR‐individuals and in blue for HC. Confidence intervals that does not cross zero indicates cognitive functions which contributes reliably to the pattern. If a bar turns upward from zero, the cognitive function is positively correlated to the pattern of white matter saliences, and is negative correlated if turning downward. Cognitive test where a lower score is better were reversed for the PLS‐C analysis. For identifying the labels of the 48 ROIs displayed by numbers (1–48), see Text S2. Abbreviations: FA, fractional anisotropy; HCs, healthy controls; JHU, John Hopkins University; LV, latent variable; ROI, region of interest; UHR, ultra‐high risk of psychosis; WM, white matter [Color figure can be viewed at http://wileyonlinelibrary.com]

LV1 comprised a pattern of higher FA in 47 out of 48 ROIs. This pattern of widespread higher FA was positively associated with a pattern of better cognitive performance in 7 out of 16 subtests for UHR‐individuals (verbal IQ and fluency, processing speed, strategies in spatial working memory, cognitive flexibility, visual learning and memory, and sustained attention). For HC, the pattern of widespread higher FA was associated with better performance on one subtest (spatial working memory) and worse performance on two subtests (verbal learning, memory, and sustained attention) (Figure 3).

To validate the primary result, we next performed a within‐group PLS‐C analysis. In the UHR‐group, PLS‐C correlation analysis identified differential associations between a pattern of regional FA and cognitive functions (omnibus test *p* = .0043). One significant latent variable (LV1) explained 60% of the covariance (*p* = .0012), revealing a similar association between widespread higher regional FA and better cognitive functions as described in the between‐group test (see Figure S3). When testing HC, PLS‐C analysis could not identify any associations between patterns of regional FA and cognitive functions (omnibus test *p* = .26) and no significant LVs. Furthermore, the posthoc test of the contribution of general intelligence did not change the result of the primary analysis (see Text S3 for details).

**Figure 4:**
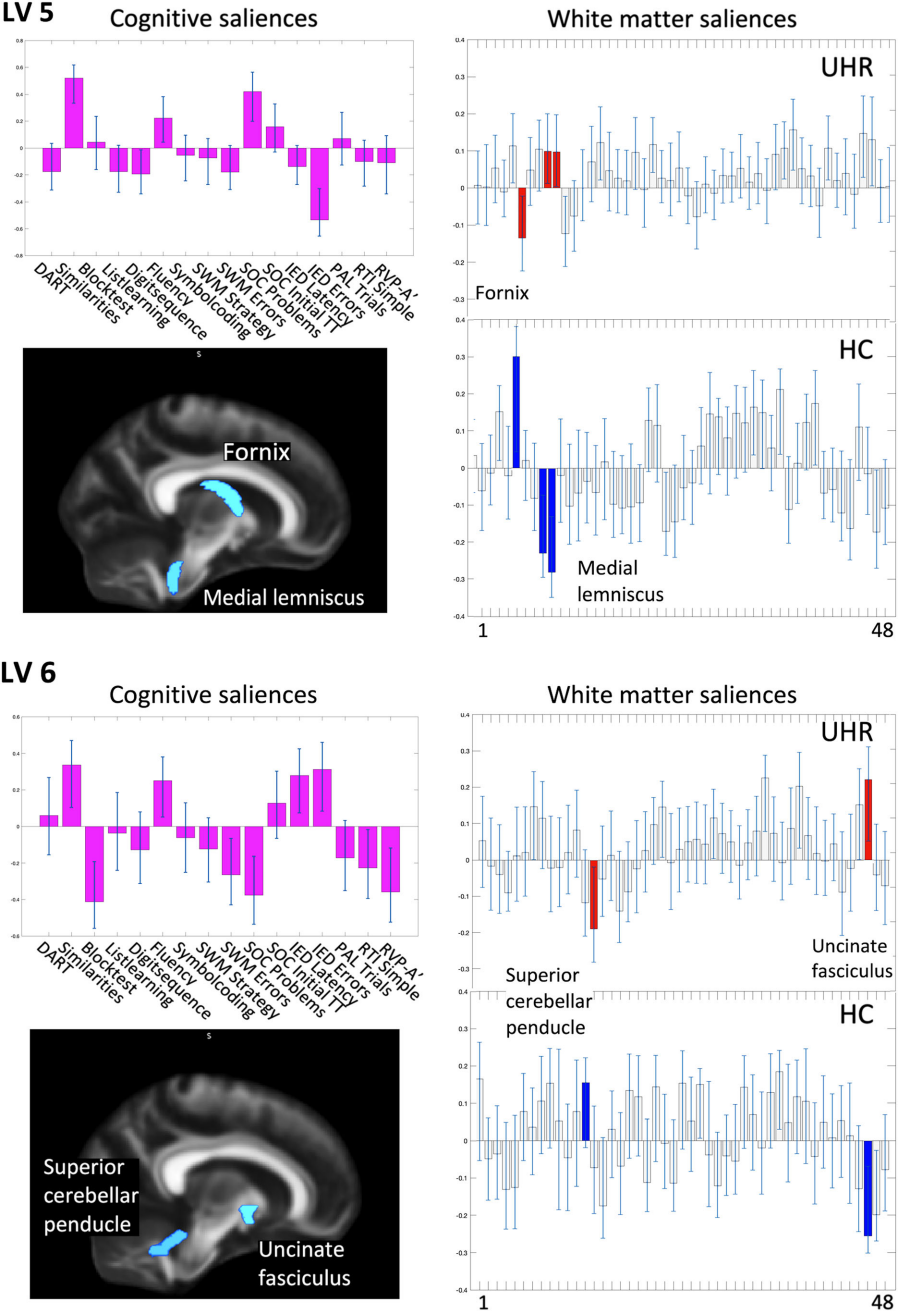
Secondary PLS‐C interaction analysis. The results from the secondary PLS‐C analysis, illustrating LV5 and LV6. Left column displays the saliences of the 16 cognitive functions in purple bars. Cognitive test where a lower score is better were reversed for the PLS‐C analysis. Confidence intervals are marked with light‐blue lines in each bar. Confidence intervals that do not cross zero contribute reliably to the pattern. If a bar turns upward from zero, the cognitive function is positively correlated to the pattern of white matter saliences, and is negative correlated if turning downward. Below, the regions with significant interaction‐effect are projected on a standard brain derived from JHU WM‐atlas. In the left column, FA‐saliences of the 48 ROIs are displayed in gray bars and stacked for each group separately. Regions with interaction‐effect are highlighted in red for UHR‐individuals and in blue for HC. For identifying the labels of the 48 ROIs displayed by numbers (1–48), see Text S2. Abbreviations: FA, fractional anisotropy; HCs, healthy controls; JHU, John Hopkins University; LV, latent variable; ROI, region of interest; UHR, ultra‐high risk of psychosis; WM, white matter [Color figure can be viewed at http://wileyonlinelibrary.com]

The secondary between‐group PLS‐C interaction analysis revealed significant correlations between a pattern of cognitive functions and different patterns of regional FA in UHR‐individuals compared to HC (omnibus test *p* = .038). Two significant latent variables (LVs) were identified: LV5 (*p* = .002) explained 7% of the covariance, and LV6 (*p* = .011) explained 5% of the covariance (Figure 4). Results from the nonsignificant latent variables LV1‐LV4 are presented in Table S7.

LV5 comprised a pattern of cognitive functions characterized by higher scores on verbal IQ, verbal fluency, planning, and poorer function on verbal working memory and more latency in cognitive flexibility. This pattern of cognitive functions was associated with a localized interaction‐effects on FA for UHR‐individuals versus HC in fornix and medial lemniscus bilaterally. Thus, the pattern of cognitive function was associated with lower FA in the fornix for UHR‐individuals, and with higher FA for HC. Conversely, this pattern of cognitive function was associated with higher FA in medial lemniscus for UHR‐individuals and lower FA for HC (Figure 4).

LV6 comprised a pattern of cognitive functions characterized by higher scores on verbal IQ, verbal fluency, cognitive flexibility including latency, and with poorer function on performance IQ, spatial working memory, planning, reaction time and sustained attention. This pattern of cognitive functions was associated with localized interaction‐effects on FA for UHR‐individuals versus HC in the left uncinate fasciculus and left superior cerebellar peduncle. Thus, the pattern of cognitive function was associated with higher FA in left uncinate fasciculus for UHR‐individuals, and lower FA for HC. Conversely, the pattern of cognitive function was associated with lower FA in left superior cerebellar peduncle for UHR‐individuals, and higher FA for HC (Figure 4).

### 3.3 Exploratory analyses on additional WM‐indices

The exploratory between‐group ANCOVA revealed highly significant interaction‐effects for all WM‐indices (FA, AD, RD, and MD) in the fornix. For the left medial lemniscus and left uncinate fasciculus the analyses revealed highly significant interaction‐effects for FA and RD, while there was only an interaction‐effect found for FA for the superior cerebellar peduncle (Table 3).

**Table 3:** ANCOVA on white matter‐indices

		FA	AD	RD	MD
LV5					
Fornix	*p*‐value	<.01^a^	<.01^a^	<.01^a^	<.01^a^
	*F*‐value	12.77	18.06	14.62	6.27
Right medial lemniscus	*p*‐value	.01^a^	.61	.02^a^	.19
	*F*‐value	6.29	0.25	5.73	1.69
Left medial lemniscus	*p*‐value	<.01^a^	.58	<.01^a^	.13
	*F*‐value	7.84	0.31	7.31	2.37
LV6					
Left uncinate fasciculus	*p*‐value	<.01^a^	.14	.01^a^	.39
	*F*‐value	8.00	2.18	6.56	0.73
Left superior cerebellar peduncle	*p*‐value	<.05^a^	.51	.10	.37
	*F*‐value	4.04	0.45	2.66	0.81

*Note*: Table 3 shows the results from the exploratory interaction test on additional white matter‐indices. The regions tested had demonstrated interaction‐effect in the PLS‐C, and next we performed analyses of covariance (ANCOVA) by correlating cognition scores with fractional anisotropy (FA) and the additional WM‐indices: axial diffusivity (AD), radial diffusivity (RD), and mean diffusivity (MD).

Abbreviations: FA, fractional anisotropy; LV, latent variable; PLS‐C, partial least square correlation; WM, white matter.

a Significant interaction‐effect. Slopes are visualized in Table S5.

## 4 DISCUSSION

In this cross‐sectional study, the univariate GLM‐analyses revealed expected impairments on multiple cognitive functions in UHR‐individuals compared to HC. The results of the voxel‐based analyses also aligned with previous reports on subtle WM‐alterations in UHR‐individuals (Krakauer et al., 2018). The small cluster we identified, was not localized exclusively in one WM‐region, but comprised right SLF and right cingulate gyrus (CG). The localization of the FA‐abnormalities is in line with previous studies identifying altered WM‐microstructure in UHR‐individuals (Clemm Von Hohenberg et al., 2014; Krakauer et al., 2017) and patients with schizophrenia (Hatton et al., 2014) and affective disorders (Dong et al., 2017). Our ROI‐analysis confirmed the finding of lower FA in SLF in UHR‐individuals compared to HC. Interestingly, the integrity of SLF has been associated with language functions (Hua et al., 2009). One functional MRI study in UHR‐individuals has demonstrated dysconnectivity in areas thought to be involved in language comprehension, strongly connected via the SLF (Jung et al., 2012). This corresponds to our results on cognitive functions, displaying particularly large effect‐sizes in the between‐group test of verbal functions (learning, memory, and fluency). We speculate, that these impairments in verbal functions may be related to the reduced FA in SLF in UHR‐individuals.

Using multivariate PLS‐C, we identified differential associations between a pattern of regional FA and cognitive functions comparing UHR‐individuals to HC. The result indicates widespread higher FA to be strongly linked to better performance on a range of cognitive functions for UHR‐individuals, but not for HCs. The cognitive performance of UHR‐individuals appears in contrast to HC more dependent on WM‐characteristics, and the comprehensive reductions in cognitive functions combined with the subtle WM‐changes identified in the univariate testing, suggest the cognitive functions in individuals at UHR to be more susceptible to variations in WM compared to HC. We speculate, if the result may reflect a difference in the ability to adjust and compensate for local FA‐alterations. This would correspond to the notion of cognitive reserve (Stern, 2009), which is a concept that has been proposed to describe differences in vulnerability and resilience to for example, structural brain alterations (such as due to pathology, aging or environmental stimuli [Barnett, Salmond, Jones, & Sahakian, 2006]), in part reflecting the capacity and efficiency of brain‐processing. Aspects of cognitive reserve are thought to involve adaptive and compensatory functions, and cognitive reserve is related to the overlapping concept of brain reserve (Satz, Cole, Hardy, & Rassovsky, 2011), and may involve flexible‐hub systems (Schmidt et al., 2015), dynamic network theory (Medaglia, Pasqualetti, Hamilton, Thompson‐Schill, & Bassett, 2017), and connectivity (Friston, 1994). Reduced cognitive reserve may help explain the discrepant link between the subtle WM‐alterations and the widespread impact on cognitive functions, as the ability to activate compensatory mechanisms and strategies to the structural alterations could be diminished. As structural neuroplasticity has been suggested to respond to training‐mediated activation (Kristensen et al., 2018), the implications regarding psychological interventions targeting compensatory cognitive training may be promising, potentially contributing to an increase in cognitive reserve.

The identification of aberrant associations between patterns of white‐matter organization and cognitive function was strengthened by the second PLS‐C interaction analysis, as we further identified two latent variables, displaying cognitive functions associated with different patterns of regional FA, showing group interaction‐effects in fornix, medial lemniscus, left superior cerebellar peduncle, and left uncinate fasciculus. The result suggests, that the underlying WM organization associated to the patterns of cognitive functions are different in UHR‐individuals compared to HC, and that specific regions drive this difference. We note that across the uni‐ and multivariate tests, specific cognitive functions appear more susceptible. The univariate test revealed the largest effect‐sizes on reduced cognitive function for UHR‐individuals in verbal memory, learning and ‐fluency, as well as processing speed and sustained attention. In the multivariate testing, these cognitive functions were all contributing reliably to the patterns of covariation between WM and cognition. Thus, these cognitive functions appear specifically affected in UHR‐individuals, and concurrently associated to structural WM alterations.

WM‐alterations in fornix have been demonstrated in studies of patients with schizophrenia (Knöchel, Schmied, & Oertel‐Knöchel, 2016; Thomas, Koumellis, & Dineen, 2011). As a main hippocampal output pathway, the involvement of fornix in especially memory functions is well supported (Hodgetts et al., 2017; Kehoe et al., 2015). However, due to its proximity to the ventricular system the fornix is particularly susceptible to cerebrospinal fluid‐induced volume effects on WM FA (Kaufmann et al., 2017), and the results should be interpreted with caution. The medial lemniscus is a main pathway of neural fibers from the cerebellum and is considered a cerebello‐thalamo‐cortical connection (CTC; Kamali, Kramer, Butler, & Hasan, 2009). Left superior cerebellar peduncle is also considered a thalamo‐cortical connection (Voogd & Baarsen, 2013), previously found to be aberrant in patients with schizophrenia (Thomason & Thompson, 2011). CTC‐connectivity has been associated with symptom course in UHR‐individuals, suggesting it may be a biomarker of disease progression (Bernard, Orr, & Mittal, 2017). Cumulative evidence has suggested that cerebello‐cortical disconnections may play a major role in the appearance of cognitive dysfunctions in patients with schizophrenia (Andreasen & Pierson, 2008; Lekeu et al., 2002), while mediating the involvement of cerebellum in higher‐order cognitive processes such as planning, verbal fluency, mental flexibility, abstract reasoning, and working memory (Wang et al., 2003). Thus, our results add further support for involvement of CTC‐connections in higher cognitive processes, which are susceptible to disease‐specific impairments.

WM‐alterations in the uncinate fasciculus have been demonstrated in studies of patients with schizophrenia and schizotypal personality disorder (Kawashima et al., 2009; Kubicki et al., 2002). In addition, the left uncinate fasciculus has been associated with IQ, verbal and visual memory, executive function (Gurrera et al., 2007; Nakamura et al., 2005), and language functions in schizotypal personality disorder (Rodrigo et al., 2007). The uncinate fasciculus is part of the limbic system and therefore implicated in emotion formation and processing. We speculate, if our results could reflect that a majority of the UHR‐individuals were diagnosed with co‐morbid affective and/or anxiety disorders. This would be in line with the results from a recent meta‐analysis on WM‐alterations across emotional disorders (affective and anxiety disorders), showing transdiagnostic communalities in WM‐tracts, with the uncinate fasciculus contributing significantly (Jenkins et al., 2016). For the uncinate fasciculus as well as for medial lemniscus, the analysis of covariance revealed a significant group interaction‐effect for FA and RD, but not for AD and MD. Changes in FA explained by interaction with RD has been linked to myelination (Table S6). Thus, dysmyelination may in part explain the aberrant FA for UHR in left uncinate fasciculus and medial lemniscus. Dysmyelination has been found to be a prominent feature of WM‐alterations in schizophrenia (Flynn et al., 2003; Seal et al., 2008).

The multivariate associations between patterns of cognitive performance and the interaction‐effect of the directionality of the regional FA identified by the PLS‐C, was confirmed in the explorative ANCOVA‐analysis. The directionality (lower vs. higher) of the FA‐alterations associated with the patterns of cognitive functions were heterogenous and varied between regions. Although the primary PLS‐C analysis associated widespread higher FA with better cognitive function, this was not the case for specific regions identified in the secondary PLS‐C analysis. It has previously been demonstrated, that the meaning of the directionality of FA is specific to the patient sample and region of interest (Thomason & Thompson, 2011). The interpretation of directionality is complex (Hoeft et al., 2007), as regions characterized with higher FA has been proposed to possibly reflect compensatory mechanisms to disease progress (Di Biase, Cropley, Cocchi, Fornito, & Calamante, 2018), or lower FA reflecting disease‐specific processes such as demyelination (Haroutunian et al., 2014). The localization of lower FA in SLF in the univariate voxel‐wise and ROI analyses could indicate another perspective regarding WM‐maturation, as the SLF in a meta‐analysis on normal development of FA has been identified as one of the most dynamically changing WM tracts during adolescence and early adulthood (Peters et al., 2012). A central developmental theory on WM‐alterations explains, how altered regional FA could be a result of aberrant developmental processes (e.g., abnormal pruning), causing different age‐trajectories in WM‐maturation (Kochunov et al., 2012; Pantelis et al., 2005). Unfortunately, our study design does not allow us to further disentangle the complex associations between cognition and WM.

The lack of correspondence between the results for regional WM‐abnormalities in the uni‐ and multivariate analyses, further illustrates the complexity of higher‐order cognitive functions relying on multiple cerebral functional and structural prerequisites. These results underscore that the application of complementary methods of analysis provide more complete information, revealing different aspects of the WM‐alterations in UHR‐individuals. Further multivariate and multimodal studies are needed to identify the functional implications, and the biological processes underlying specific combinations of WM‐indices.

An apparent strength of our study is the large sample‐size of 116 UHR‐individuals and 49 HC. Also, the uni‐ and multivariate statistical methods were complementary in identifying WM‐alterations. In particular, PLS‐C allowed for the investigation of the complex associations between cognitive function and WM.

Some limitations should be considered. Overall, the results of PLS‐C can be difficult to interpret due to the complexity of covarying multiple data. Nonetheless, we find the results from our study to be both informative and supportive of recent findings and conceptualizations in schizophrenia research. Another methodological limitation is due to the skeleton‐based analyses of regional FA, which only estimates the characteristics of the underlying fiber‐tracts.

Secondly, the UHR‐individuals are a diagnostically heterogenous and complex group, and the rates of transition to psychosis for our participants is still unknown. Our patient sample may therefore be regarded as a mixed help‐seeking clinical population, rather than a specific prodromal group prior to their onset of frank psychosis. Furthermore, only UHR‐individuals from the age of 18 could be included due to the organizational distinction between adult versus child‐ and adolescent psychiatry in Denmark. We also included UHR‐individuals with substance abuse‐ and dependency to ensure the large sample‐size and the external validity. However, we attempted to account for this limitation by testing the effect of both medication and substance abuse‐ and dependency posthoc. Finally, the difference in sample‐size between UHR‐individuals and HC may reduce statistical power in the between‐group analyses.

In conclusion, our univariate analyses confirmed the expectation that UHR‐individuals displayed subtle WM‐alterations and impaired cognition. The multivariate PLS‐C analyses identified differential associations between patterns of cognitive function and WM in UHR‐individuals compared to HC. Widespread higher FA was strongly linked to better cognitive performance for UHR‐individuals, but not for HCs. The involvement of specific cerebello‐thalamo‐cortical‐connections in higher cognitive processes was supported, and the altered associations between cognitive functions and WM may be explained by underlying region‐specific dysmyelination in UHR‐individuals. Longitudinal studies are required to determine if these cross‐sectional associations between cognition and WM microstructure are static, or change over time depending on the symptom course or clinical interventions.

## Supporting information


**Table S1** Cognitive functions by outcome measures
**Table S2**. Image quality metrics
**Table S3**. Group differences on regional FA
**Table S4** Univariate GLM on regional FA
**Table S5**. Exploratory ANOCOVA interaction analysis
**Table S6**. Biological processes underlying combinations of WM‐indices
**Table S7**. Non‐significant latent variables LV1–LV4
**Figure S1**. FA‐skeleton maps and white matter regions
**Figure S2**. Permutation test for between‐group interaction analysis
**Figure S3**: PLS‐C within‐group analysis
**Figure S4** Plot of FA as a function of age
