# Supplementary material for: Widespread higher fractional anisotropy associates to better cognitive functions in individuals at ultra‐high risk for psychosis
Source: Hum Brain Mapp. 2019 Aug 20;40(18):5185–201. doi: 10.1002/hbm.24765 (PMC6864899; doi:10.1002/hbm.24765)
Supplement: Supplementary file 1 — Table S1 Cognitive functions by outcome measures Table S2. Image quality metrics Table S3. Group differences on regional FA Table S4 Univariate GLM on regional FA Table S5. Exploratory ANOCOVA interaction analysis Table S6. Biological processes underlying combinations of WM‐indices Table S7. Non‐significant latent variables LV1–LV4 Figure S1. FA‐skeleton maps and white matter regions Figure S2. Permutation test for between‐group interaction analysis Figure S3: PLS‐C within‐group analysis Figure S4 Plot of FA as a function of age [file HBM-40-5185-s001.doc]

**Table S1: Cognitive functions by outcome** measures

| **Cognitive function** | **Test** | **Outcome measure** |
| --- | --- | --- |
| Premorbid verbal IQ | (DART)  Danish Adult Reading Test | - Number of words correctly pronounced |
| Current Verbal IQ | (WAIS-III)  Similarities subtest of Wechsler Adult Intelligence Scale | - Total score, i.e. defining a conceptual similarity between two words. |
| Current performance IQ | (WAIS-III)  Block design subtest of Wechsler Adult Intelligence Scale | - Total score, i.e. patterns correctly arranged within the time-limit. |
| Learning and memory | BACS  List learning | - Total number of words correctly recalled across 5 trials. |
| Verbal working memory | BACS  Digit sequencing | - Number of correctly ordered sequences of digits. |
| Verbal fluency | BACS  Verbal fluency, semantic and phonetic | - Number of correct words generated (supermarket items, F- and S-words). |
| Processing speed | BACS  Symbol coding | - Number of written symbols correctly matched with numbers. |
| Spatial working memory | CANTAB (SWM)  Spatial Working Memory | - Total errors. - Strategy scores, i.e. the efficient use of a search strategy. |
| Planning | CANTAB (SOC)  Stockings of Cambridge | - Problems solved in minimum moves. - Mean initial thinking time (5 moves), i.e. the time used planning for 5 move problems. |
| Set shifting/mental flexibility | CANTAB (IED)  Intra-Extra-Dimensional Set-shifting | - Total errors, adjusted for the number of stages not completed. - Total latency. |
| Visual memory and learning | CANTAB (PAL)  Paired Associated Learning | - Total errors adjusted for stages not attempted. - Total trials adjusted for stages not completed. |
| Reaction time | CANTAB (RTI)  Reaction Time | - Mean simple reaction time. |
| Sustained Attention | CANTAB (RVP-A’)  Rapid Visual Processing | - Signal detection (a’), i.e. the sensitivity in detecting targets, regardless of response tendency. |

Table S1 shows the categories of cognitive functions associated with the specific tests and outcome measures applied in the analyses.

**Table S2. Image quality metrics**

|  | **TSNR**  **Mean (SD)** | **MAXVOX**  **Mean (SD)** | **MEANVOX**  **Mean (SD)** |
| --- | --- | --- | --- |
| UHR-individuals and HCs in the full sample (N=165) | 6.98  (0.36) | 4539.6  (6203) | 863.8  (431.3) |
| Roalf et al. (N=147)  “Poor” | 5.52  (0.93) | 14497  (8667) | 2001.50  (1080.20) |
| Roalf et al. (N=468)  “Good” | 6.9  (0.68) | 7165  (7189) | 830.40  (597.10) |
| Roalf et al. (N=742)  “Excellent” | 7.37  (0.55) | 1684  (1741) | 378  (164.10) |

Table S2 shows mean (SD) of quality metrics on DWI data for the UHR-individuals and healthy controls in the full sample, as well as for the quality assessment groups defined by Roalf et al. (Roalf et al., 2016). Quality control was done by visually inspecting all DW images slice by slice before processing, and excluded if the image quality was judged to be of poor quality. Three image quality metrics (temporal signal-to-noise ratio (TSNR), maximum voxel intensity outlier count (MAXVOX) and mean voxel intensity outlier count (MEANVOX)) were calculated from each subjects DW image using a quality assessment method described in Roalf et al. The measured quality metrics in this study ranged between the ‘good’ and ‘excellent’ quality.

Abbreviations: DWI: diffusion weighted imaging; FA: fractional anisotropy; HC: healthy controls; SD: standard deviation; UHR: ultra-high risk

**Table S3. Group-differences on regional FA**

| **ROI** | **UHR-individuals**  **Mean FA (S.D.)**  (N=116) | **Healthy controls**  **Mean FA (S.D.)**  (N=49) | ***p-*value**  **Effect size**  (Hedges’ *g)* |
| --- | --- | --- | --- |
| Right anterior corona radiata | 0.4868 (0.0239) | 0.4980 (0,0256) | P=0.02 *g*=0.46 |
| Right fornix (cres) stria terminalis | 0.5734 (0.0311) | 0.5870 (0,0259) | P=0.04 *g*=0.46 |
| Right superior longitudinal fasciculus | 0.5355 (0.0273) | 0.5468 (0,0224) | P<0.05 *g*=0.43 |
| Left tapetum | 0.6469 (0.0625) | 0.6673 (0,0626) | P<0.05 *g*=0.33 |

Table S3 displays the results from the univariate GLM between-group analysis on regional fractional anisotropy (FA). The four out of 48 ROIs with significant group-effect is reported, all characterized by lower FA in the UHR-individuals compared to healthy controls. Bonferroni corrected significance level was calculated to p<0.001.

Abbreviations: GLM: general linear modelling; ROI: region of interest; SD: standard deviation; UHR: ultra-high risk

**Table S4 Univariate GLM on regional FA**

|  | **Antipsychotic-naïve** (N=67 UHR / 49 HC) | **Psychotropic medication-naïve** (N=31 UHR / 49 HC) | **Psychotropic-medication, and substance-abuse and dependency-naïve** (N=UHR 28 / 49 HC) |
| --- | --- | --- | --- |
| **Right superior longitudinal fasciculus** | P=0.031 | P=0.450 | P=0.432 |
| UHR FA Mean (SD) | 0.534 (0.027) | 0.542 (0.022) | 0.542 (0.025) |
| HC FA Mean (SD) | 0.547 (0.022) | 0.547 (0.024) | 0.547 (0.022) |
| **Right cingulate gyrus** | P=0.760 | P=0.769 | P=0.744 |
| UHR FA Mean (SD) | 0.596 (0.033) | 0.594 (0.030) | 0.595 (0.030) |
| HC FA Mean (SD) | 0.602 (0.031) | 0.602 (0.031) | 0.601 (0.031) |
| **Right anterior corona radiata** | P=0.009 | P=0.293 | P=0.251 |
| UHR FA Mean (SD) | 0.486 (0.023) | 0.488 (0.024) | 0.487 (0.024) |
| HC FA Mean (SD) | 0.498 (0.026) | 0.498 (0.026) | 0.498 (0.026) |
| **Right fornix (cres) stria terminalis** | P=0.031 | P=0.113 | P=0.145 |
| UHR FA Mean (SD) | 0.573 (0.029) | 0.577 (0.023) | 0.578 (0.023) |
| HC FA Mean (SD) | 0.587 (0.026) | 0.587 (0.026) | 0.587 (0.026) |
| **Left tapetum** | P=0.063 | P=0.380 | P=0.289 |
| UHR FA Mean (SD) | 0.645 (0.064) | 0.654 (0.055) | 0.651 (0.051) |
| HC FA Mean (SD) | 0.667 (0.063) | 0.667 (0.063) | 0.667 (0.063) |
| **Fornix** | P=0.652 | P=0.912 | P=0.914 |
| UHR FA Mean (SD) | 0.549 (0.062) | 0.556 (0.064) | 0.550 (0.065) |
| HC FA Mean (SD) | 0.556 (0.062) | 0.556 (0.062) | 0.556 (0.062) |
| **Left medial lemniscus** | P=0.946 | P=0.634 | P=0.548 |
| UHR FA Mean (SD) | 0.621 (0.035) | 0.620 (0.036) | 0.621 (0.036) |
| HC FA Mean (SD) | 0.626 (0.030) | 0.626 (0.030) | 0.626 (0.030) |
| **Right medial lemniscus** | P=0.879 | P=0.156 | P=0.118 |
| UHR FA Mean (SD) | 0.640 (0.034) | 0.641 (0.031) | 0.643 (0.029) |
| HC FA Mean (SD) | 0.643 (0.029) | 0.643 (0.029) | 0.643 (0.029) |
| **Left uncinate fasciculus** | P=0.592 | P=0.557 | P=0.394 |
| UHR FA Mean (SD) | 0.502 (0.0368) | 0.506 (0.042) | 0.511 (0.041) |
| HC FA Mean (SD) | 0.504 (0.037) | 0.504 (0.039) | 0.504 (0.037) |
| **Left superior cerebellar peduncle** | P=0.989 | P=0.126 | P=0.176 |
| UHR FA Mean (SD) | 0.644 (0.038) | 0.633 (0.031) | 0.636 (0.032) |
| HC FA Mean (SD) | 0.653 (0.033) | 0.653 (0.033) | 0.653 (0.033) |

Table S4 displays the results from the post-hoc univariate GLM between-group analysis on regional fractional anisotropy (FA), testing group effects of medication, substance abuse and dependency. We tested the ROIs with significant between-group effect comparing UHR-individuals and HC in the primary analyses, and performed three analyses comparing HCs with: a) antipsychotic-naïve UHR-individuals, b) psychotropic medication-naïve UHR-individuals (no lifetime exposure to antipsychotics, antidepressants, mood-stabilizers, and benzodiazepines), and c) combined psychotropic-medication, and substance-abuse and dependency-naïve UHR-individuals. Covariates were age, gender, parental SES, tobacco smoking, alcohol consumption, and relative and absolute movement in scanner. Bonferroni corrected significance level was calculated to p<0.005.

Abbreviations: GLM: general linear modelling; HC: healthy controls; ROI: region of interest; SD: standard deviation; UHR: ultra-high risk

**Table S5. Exploratory ANOCOVA interaction analysis**


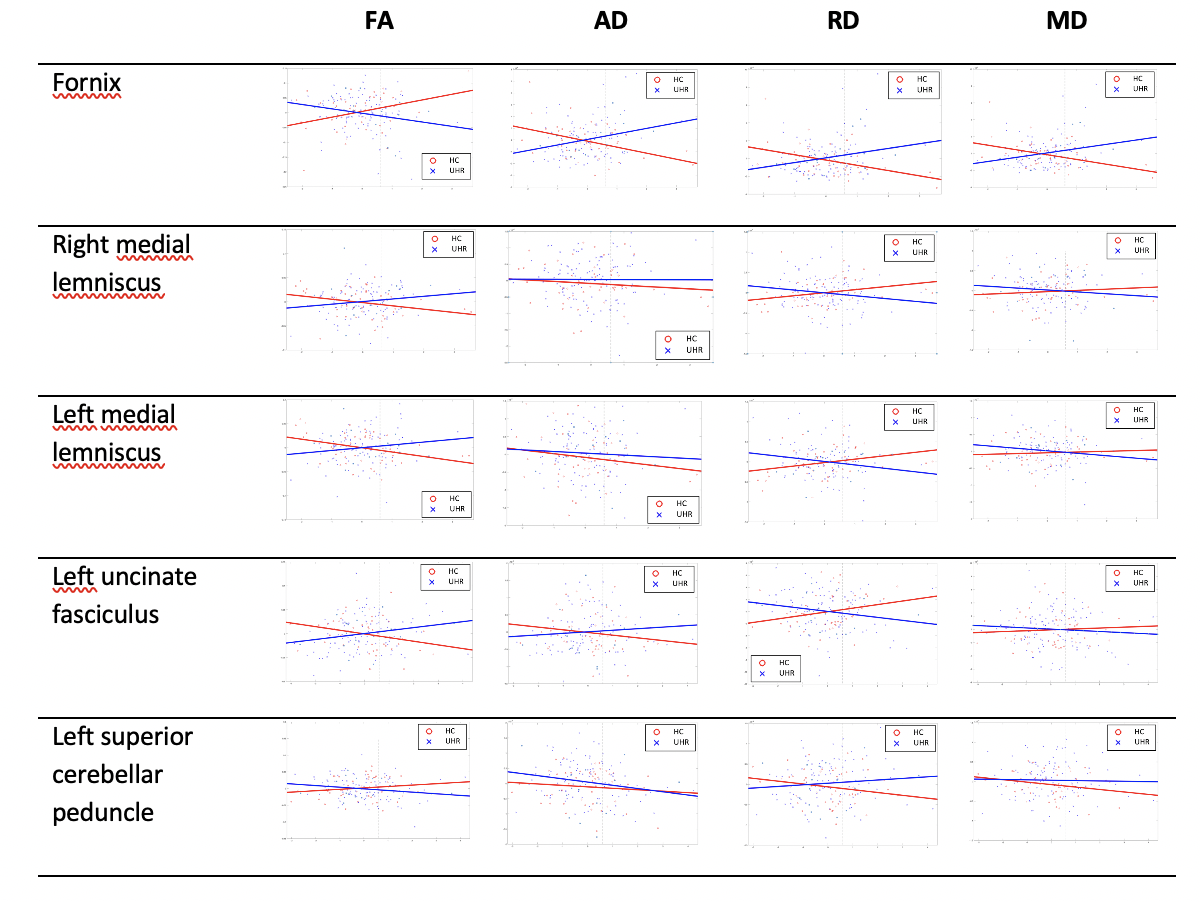


Table S5 displays the linear relations between the patterns of cognitive function of LV5 and LV6, associated with the additional white matter indices (axial diffusivity (AD), radial diffusivity (RD), and mean diffusivity (MD)) in the specific regions identified with interaction-effect in the PLS-C analysis.

Abbreviations: FA: fractional anisotropy; LV: latent variable.

**Table S6. Biological processes underlying combinations of WM-indices**

|  | **FA** | **AD** | **RD** | **MD** |
| --- | --- | --- | --- | --- |
| **Dysmyelination**  (Alexander et al., 2011; Song et al., 2002) | Reduced | Unchanged | Increased | Increased |
| **Axonal damage/loss** (Alexander, Lee, Lazar, & Field, 2007; Song et al., 2003) | Reduced | Reduced | Increased | Increased |
| **Inflammation**  (Pasternak, Sochen, Gur, Intrator, & Assaf, 2009) | Reduced |  |  | Increased |

Table S6 displays the suggested biological processes underlying the combinations of WM-indices.

Abbreviations: FA: fractional anisotropy; AD: axial diffusivity; RD: radial diffusivity; MD: mean diffusivity.

**Table S7. Non-significant latent variables LV1-LV4**

|  | **LV1** | **LV2** | **LV3** | **LV4** |
| --- | --- | --- | --- | --- |
| Explained covariance (percent) | 34.11 % | 17.18 % | 8.95 % | 8.10 % |
| Significance (P-value) | 0.74 | 0.16 | 0.50 | 0.08 |

Table S7 displays the results from the PLS-C test on the non-significant latent variables LV1-LV4. The numerical order (LV1, LV2, LV3, LV4) of the LVs is based on the portion of the covariance matrix they explain. All LVs combined completely describe the covariance matrix (100%). However, in our study we were interested in LVs, that captured the part of the covariance matrix describing the differences between UHR-individuals and healthy controls. If this difference is evident enough, then the LV is significant (capturing the part of the covariance, where UHR-individuals show a different association between cognitive functions and regional FA compared to healthy controls). Thus, the size of the LV and its significance are two unrelated measures.

Abbreviations: FA: fractional anisotropy; LV: latent variable; UHR: ultra-high risk; PLS-C: partial least square correlation analysis

**Figure S1. FA-skeleton maps and white matter regions**


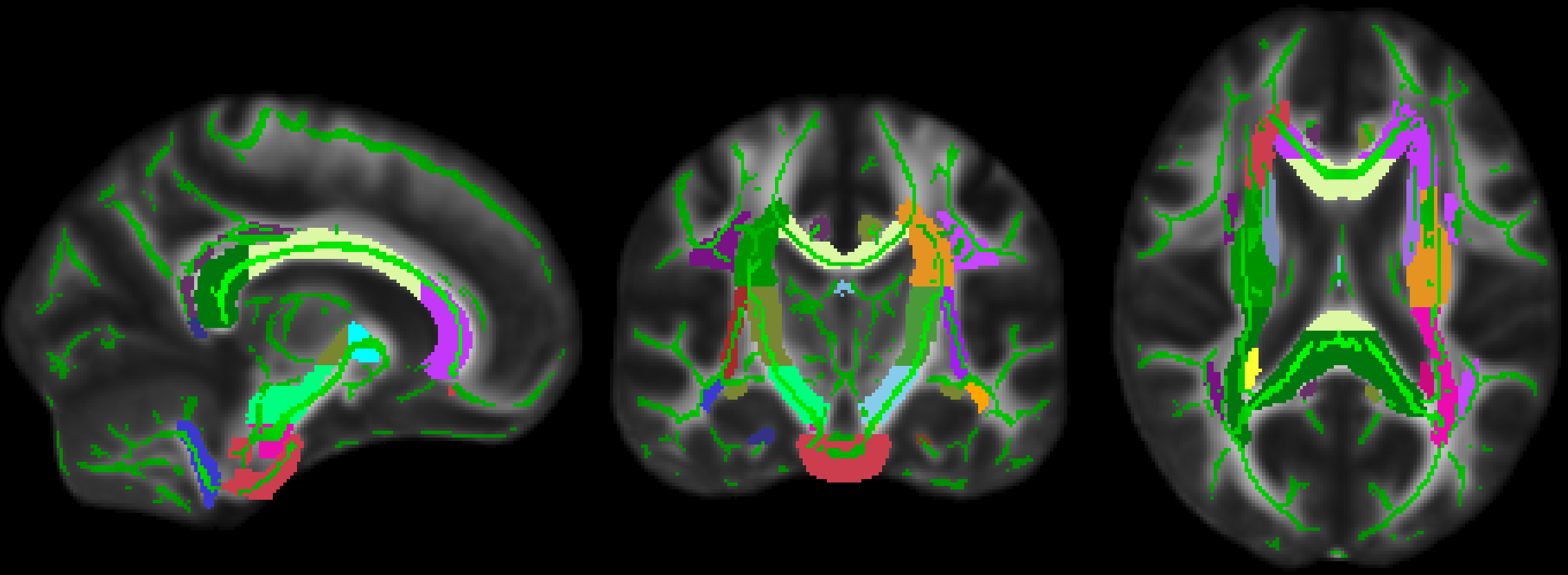


Figure S1 Illustrates a) In green the overlaid voxel-based mean FA-skeleton, aligned from participants FA-data and on a standard FA brain template. Tract-based spatial statistics was used to create the skeleton maps. The FA-skeleton is thinned, representing only the centers of FA-tracts. b) Using the FSL-JHU-DTI white-matter atlas labels, we extracted the mean FA, AD, RD and MD values in 48 WM label ROIs from the skeletonized data. Each WM-region from the atlas is displayed with a different color code.

Abbreviations: AD: axial diffusivity; DTI: diffusion tensor imaging; FA: fractional anisotropy; FSL: FMRIB Software Library; JHU: John Hopkins University; MD: mean diffusivity; RD: radial diffusivity; ROI: region of interest; WM: white matter.

**Figure S2. Permutation test for between-group interaction analysis**


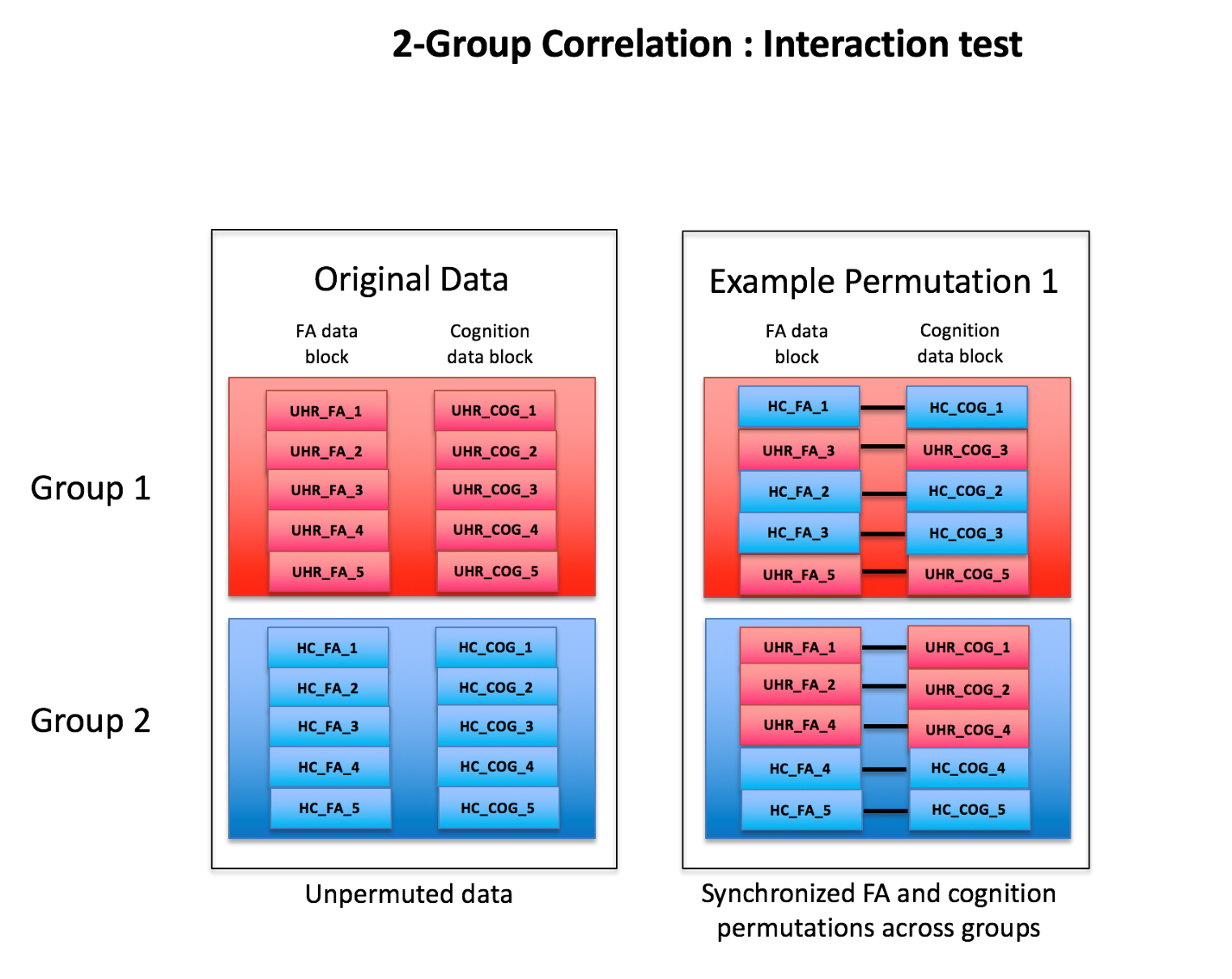


Figure S2 Illustrates the Two group interaction permutations test. The shuffling across groups during the permutations testing for interaction is performed by preserving the link between the FA and cognition (i.e. permutation order is synchronised between the FA and cognition blocks).

**Figure S3: PLS-C within-group analysis**

In the UHR-group, PLS-C correlation analysis identified differential associations between a pattern of regional FA and cognitive functions (omnibus test p=0.0043). One significant latent variable displayed below (LV1) explained 60.02% of the covariance (p=0.0012).


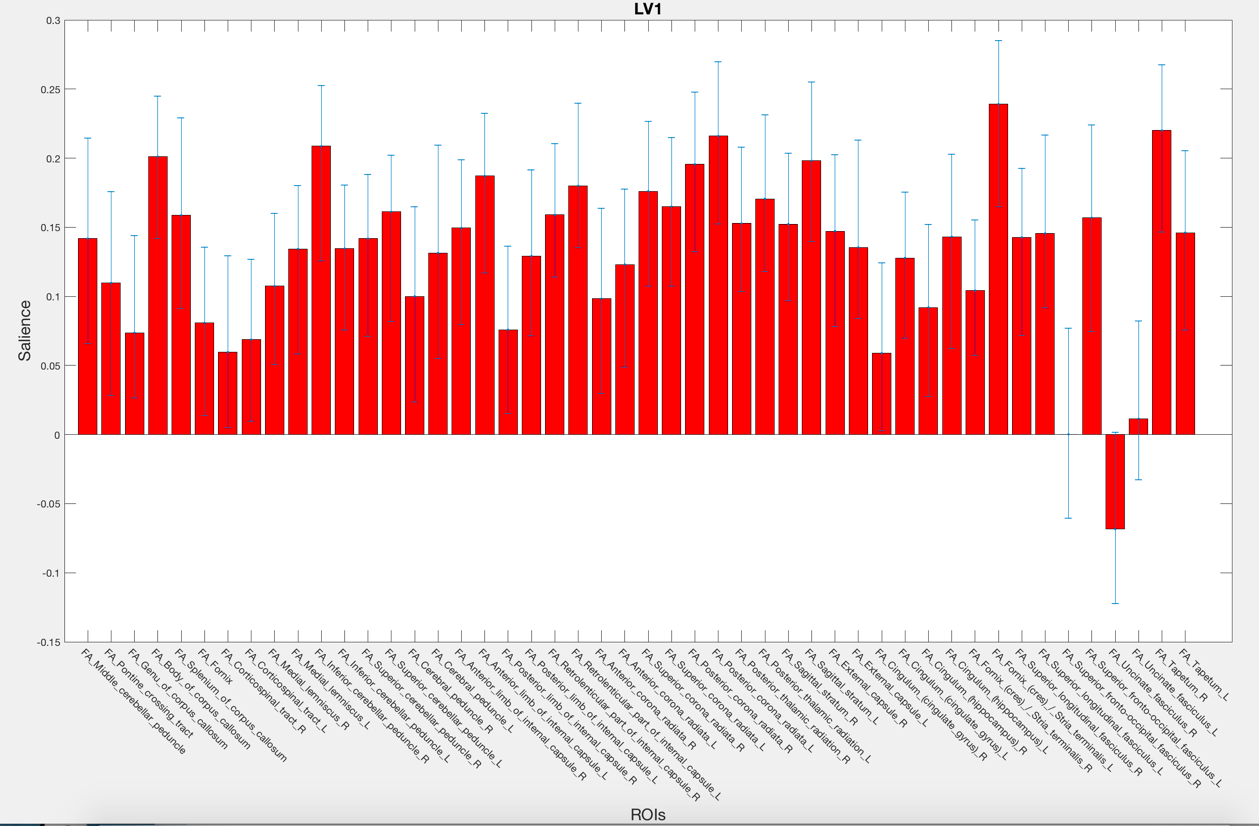

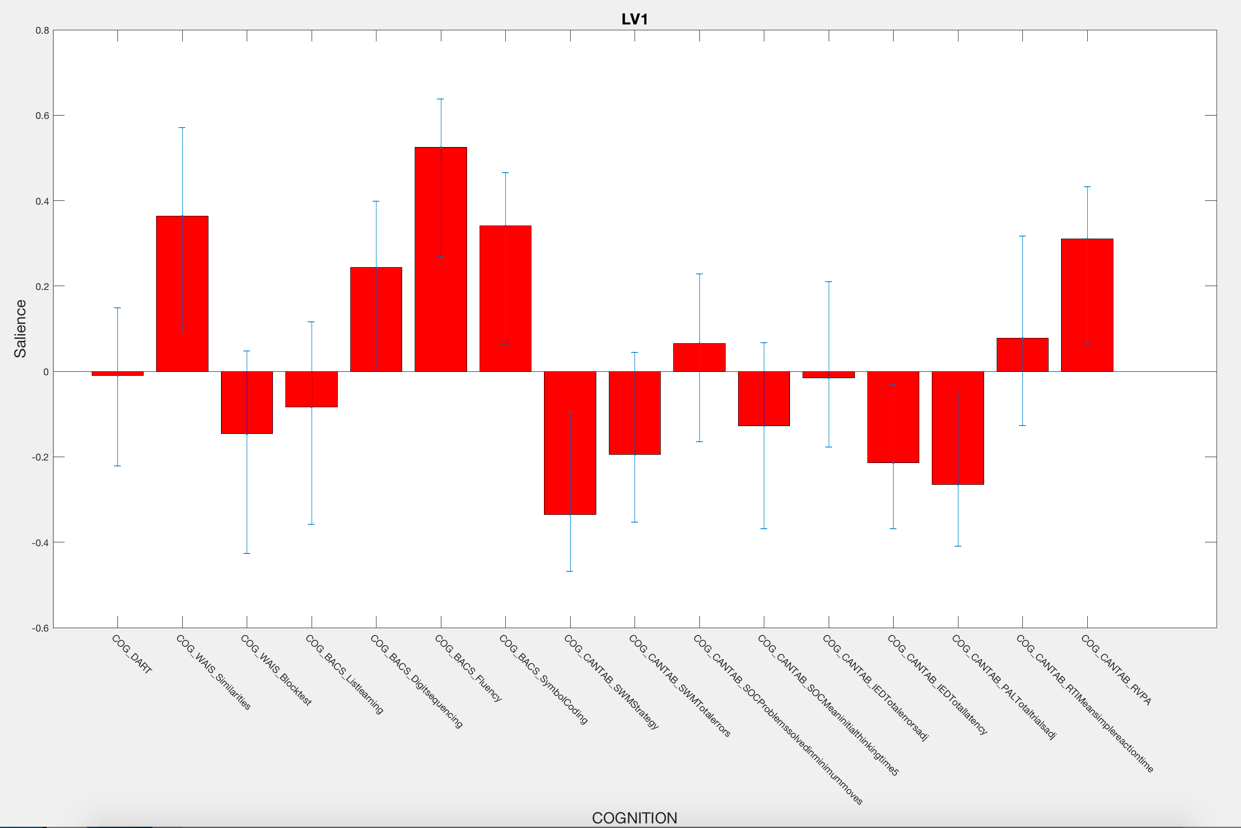


The PLS-C within-group analysis for UHR revealed a similar association between widespread higher regional FA and better cognitive functions as described in the between-group test. LV1 comprised a pattern of higher FA in 45 out of 48 ROIs. This pattern of widespread higher FA was positively associated with a pattern of better cognitive performance in 8 out of 16 subtests for UHR-individuals: verbal IQ, verbal memory and fluency, processing speed, and sustained attention, as well as the reversed subtests (lower is better): strategies in spatial working memory, latency in mental flexibility, and visual memory and learning.

**Figure S4 Plot of FA as a function of age**


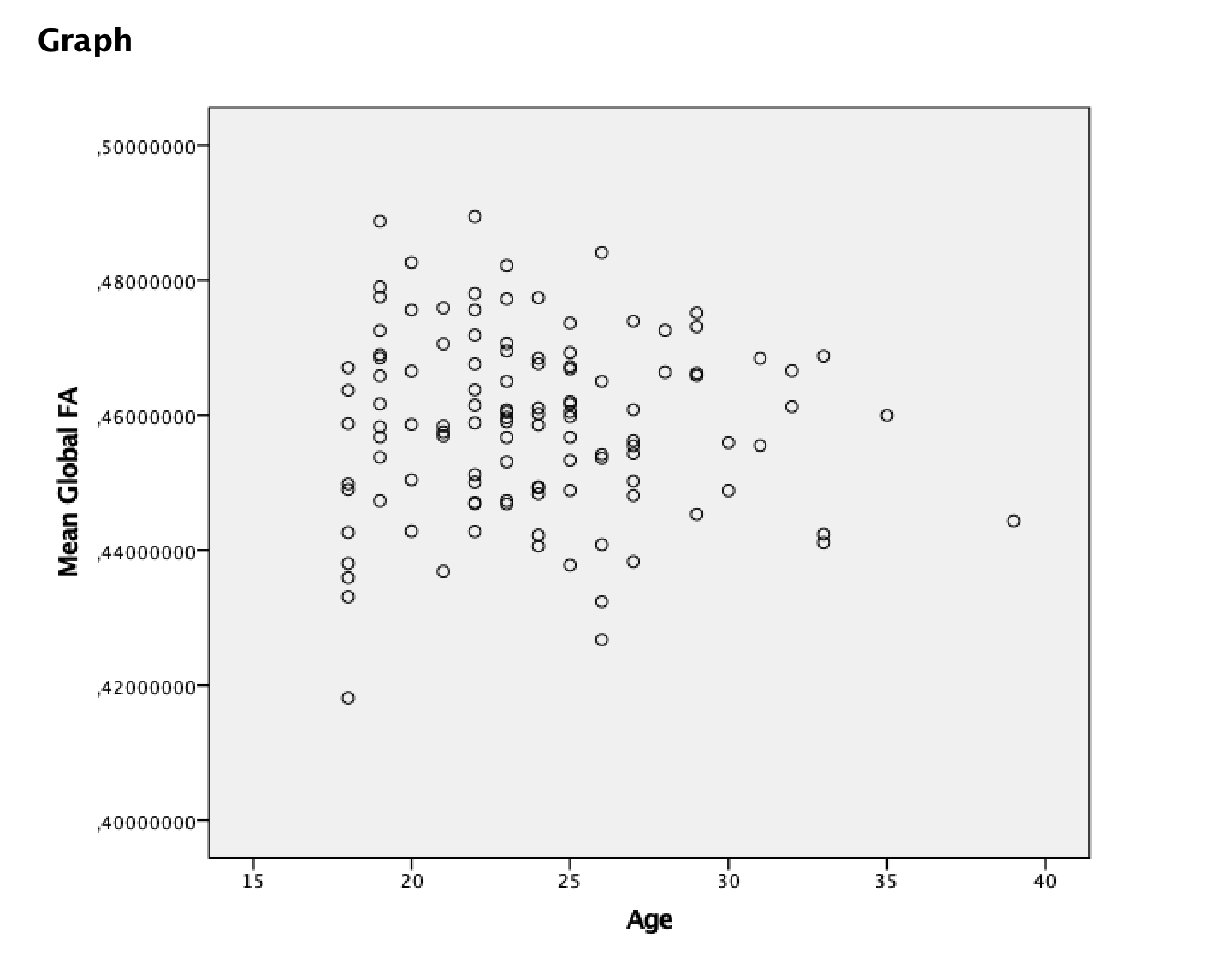

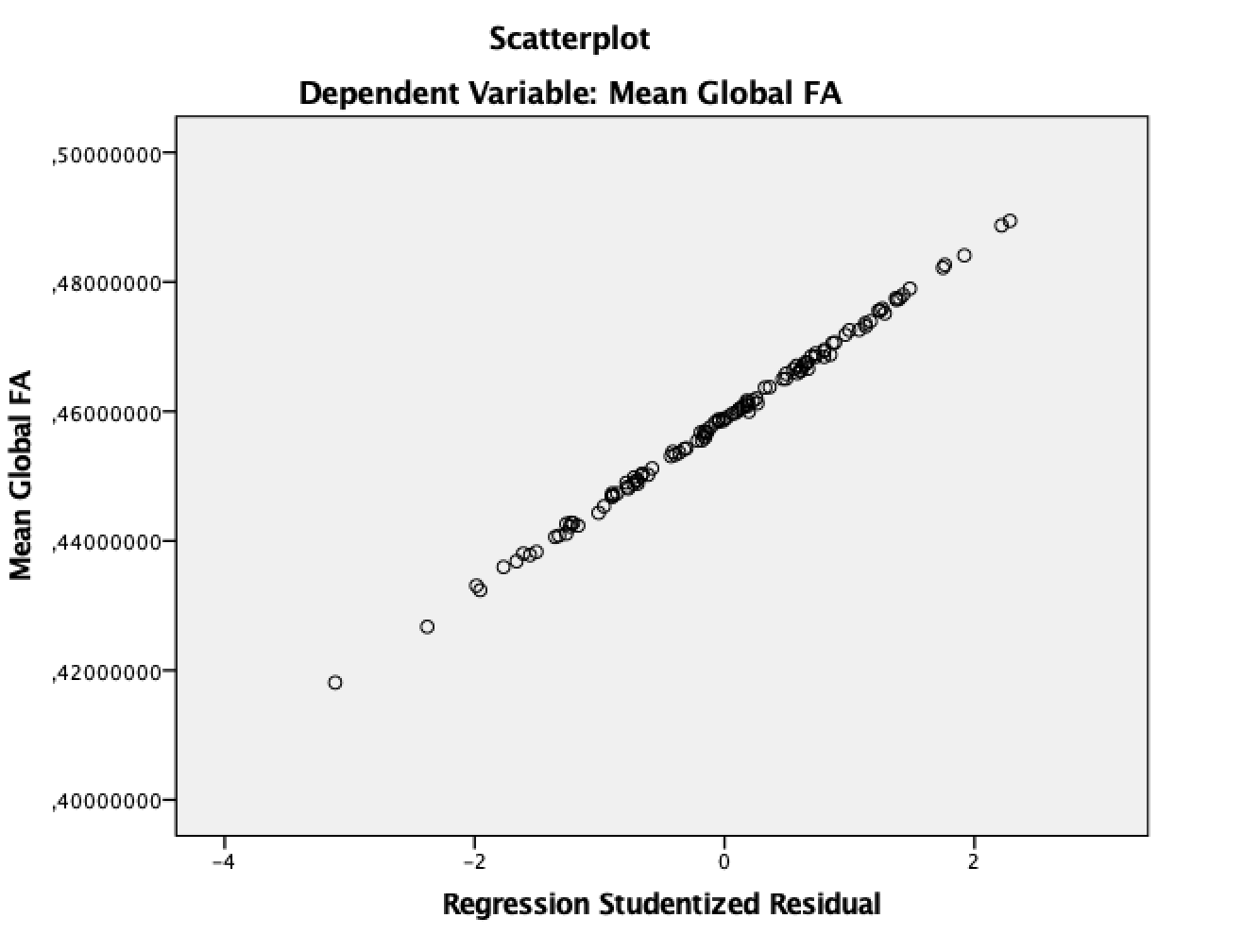


Figure S4 Illustrates the plots of a) Mean global FA as a function of age, and b) A scatterplot of Mean global FA as a function of age-residuals in a linear regression model.

Abbreviations: FA: fractional anisotropy

**Text S1. Image acquisition and processing**

Magnetic resonance imaging (MRI) scans were acquired on a 3 tesla scanner (Philips Healthcare, Best, The Netherlands), using a 32-channel SENSE head coil (Invivo, Orlando, Florida, USA). Diffusion-weighted images (DWI) were acquired using single shot spin-echo echoplanar imaging (EPI) sequence with 30 non-collinear diffusion weighted (b = 1000 s/mm^2^) scans and 5 diffusion unweighted (b = 0 s/mm^2^) scans. Other acquisition settings were: Acquisition matrix = 128 × 99 × 75; voxel dimensions = 1.88 × 2.41 × 2mm3; no slice gap=0; reconstructed voxel dimensions = 1.88 x 1.88 x 2mm^3^; TR=7058 ms; TE = 68 ms; parallel imaging SENSE factor = 3(AP); flip angle = 90°. Two DWI scans were acquired, where an opposite phase encoding direction were used in the second scan, enabling correction for susceptibility distortions (Andersson, Skare, & Ashburner, 2003; Skare & Andersson, 2001). Tools from the FSL software library v5.0.10 (Jenkinson, Beckmann, Behrens, Woolrich, & Smith, 2012) and MRtrix3 ([www.mrtrix.org](http://www.mrtrix.org/)) were used for image processing. Non-brain tissue was removed using *dwi2mask* (Dhollander, Raffelt, & Connelly, 2016). DWI data were denoised by exploiting data-redundancy in the principal component analyses (PCA) domain (Veraart, Fieremans, & Novikov, 2016; Veraart, Novikov, et al., 2016). Next, we performed B1 field inhomogeneity correction (Smith et al., 2004; Zhang, Brady, & Smith, 2001). An eddy current and susceptibility artifact correction was performed using *topup* (Andersson & Sotiropoulos, 2016) and *eddy* (Andersson & Sotiropoulos, 2016). We extracted absolute and relative head motion parameters from *eddy_resticted_movement_rms* output of *eddy*. That were used to correct for head motion in the subsequent statistical analyses. Diffusion (kurtosis) tensor were computed using rotated diffusion vectors and iterative reweighted linear least squares estimator (Veraart, Sijbers, Sunaert, Leemans, & Jeurissen, 2013). Diffusion parameter maps of FA were calculated using *tensor2metric* (Basser, Mattiello, & LeBihan, 1994). Tract-based spatial statistics (TBSS) (Smith et al., 2006, 2004) was used to align FA data using the nonlinear image registration tool (FNIRT) (Andersson, Jenkinson, & Smith, 2007a, 2007b). The resulting mean FA image (threshold of 0.2) was thinned to create a mean study-specific FA skeleton template. Next, the nearest maximum FA values of all subjects were projected onto the mean study-specific FA skeleton template (Smith et al., 2006). Subsequently, the AD, RD and MD images were non-linearly projected on the FA skeleton. Using the JHU DTI-based white-matter atlas labels (Hua et al., 2008; Mori & Zijl, 2007)**,** we extracted the mean FA, AD, RD and MD values in 48 WM label ROIs from skeletonized data. MRI quality metrics were assessed using a quality assessment method described in Roalf et al. 2016 (Table S2).

**Text S2. Labels of the 48 WM-regions**

1. Middle cerebellar peduncle

2. Pontine crossing tract

3. Genu of corpus callosum

4. Body of corpus callosum

5. Splenium of corpus callosum

6. Fornix

7. Corticospinal tract R

8. Corticospinal tract L

9. Medial lemniscus R

10. Medial lemniscus L

11. Inferior cerebellar peduncle R

12. Inferior cerebellar peduncle L

13. Superior cerebellar peduncle R

14. Superior cerebellar peduncle L

15. Cerebral peduncle R

16. Cerebral peduncle L

17. Anterior limb of internal capsule R

18. Anterior limb of internal capsule L

19. Posterior limb of internal capsule R

20. Posterior limb of internal capsule L

21. Retrolenticular part of internal capsule R

22. Retrolenticular part of internal capsule L

23. Anterior corona radiata R

24. Anterior corona radiata L

25. Superior corona radiata R

26. Superior corona radiata L

27. Posterior corona radiata R

28. Posterior corona radiata L

29. Posterior thalamic radiation R

30. Posterior thalamic radiation L

31. Sagittal stratum R

32. Sagittal stratum L

33. External capsule R

34. External capsule L

35. Cingulum cingulate gyrus R

36. Cingulum cingulate gyrus L

37. Cingulum hippocampus R

38. Cingulum hippocampus L

39. Fornix cres Stria terminalis R

40. Fornix cres Stria terminalis L

41. Superior longitudinal fasciculus R

42. Superior longitudinal fasciculus L

43. Superior frontooccipital fasciculus R

44. Superior frontooccipital fasciculus L

45. Uncinate fasciculus R

46. Uncinate fasciculus L

47. Tapetum R

48. Tapetum L

**Text S3. IQ-corrected PLS-C analyses**

Post-hoc correcting PLS-C for the effect of IQ, we tested between-group effect when excluding different measures of IQ. All tests identify only minor differences, and the result appear very consistent over several different analyses (results below).

1. Excluding DART (Premorbid IQ)

Omnibus test (p= 0.0030). LV1; cross-block covariance= 41.04 % p=0.041.

Similar association between globally higher regional FA and better cognitive functions as described in the between-group test.

2. Excluding WAIS Similarities (Current verbal IQ)

Omnibus test (p= 0.015). LV1; cross-block covariance= 41.84%, p=0.042.

Similar association between globally higher regional FA and better cognitive functions as described in the between-group test.

3. Excluding WAIS Blockdesign (Current performance IQ)

Omnibus test (p= 0.0040). LV1; cross-block covariance= 42.88%, p=0.026.

Similar association between globally higher regional FA and better cognitive functions as described in the between-group test.

4. Excluding WAIS Similarities AND Blockdesign (Current total IQ)

Omnibus test (p= 0.0110). LV1; cross-block covariance= 43%, p=0.036.

Similar association between globally higher regional FA and better cognitive functions as described in the between-group test.

5. Excluding DART AND WAIS Similarities AND Blockdesign (Premorbid and current IQ)

Omnibus test (p= 0.0020). LV1; cross-block covariance= 42.73%, p=0.042.

Similar result as described in the between-group test, but reversed association between globally lower regional FA and worse cognitive functions.

**Text S4. Conflicts of interests**

Dr. Fagerlund, Dr. Glenthøj, Dr. Jepsen, Dr. Jessen, Dr. Krakauer, MsC. Kristensen, Dr. Mandl, Prof. Nordentoft, Dr. Raghava, and MD. Wenneberg has no conflicts of interest to declare.

Prof. Glenthøj is the leader of a Lundbeck Foundation Center of Excellence for CINS (R155-2013-16337), which is partially financed by an independent grant from the Lundbeck Foundation based on international re-view and partially financed by the Mental Health Services in the Capital Region of Denmark, the University of Copenhagen, and other foundations. All grants are the property of the Mental Health Services in the Capital Region of Denmark and administrated by them.

Dr. Ebdrup has received lecture fees and/or is part of Advisory Boards of Bristol-Myers Squibb, Eli Lilly and Company, Janssen-Cilag, Otsuka Pharma Scandinavia and Takeda Pharmaceutical Company

Prof. Pantelis was supported by a grant from the Lundbeck Foundation (Grant No. R246-2016-3237) and by a Senior Principal Research Fellowship (Grant No. 1105825) from the Australian National Health and Medical Research Council.

**Text S5. Post-hoc testing the fitting of age in a quadratic model.**

As age might change non-linear with age, regressing with age could be problematic. To test, whether age would fit in a quadratic model, we performed a post-hoc univariate GLM between-group test with whole brain FA as dependent variable. In the first model, age was entered as covariate along with gender, parental SES, tobacco smoking, alcohol consumption, and relative and absolute movement in scanner. Age did not come out as significant covariate (P=0.339). In the second model, we entered age as well as age squared as nested covariate. Neither age (P=0.152) or age squared (P=0.121) came out as significant covariates.

**References**

Alexander, A. L., Hurley, S. A., Samsonov, A. A., Adluru, N., Hosseinbor, A. P., Mossahebi, P., … Field, A. S. (2011). Characterization of Cerebral White Matter Properties Using Quantitative Magnetic Resonance Imaging Stains. *Brain Connectivity*, *1*(6), 423–446. https://doi.org/10.1089/brain.2011.0071

Alexander, A. L., Lee, J. E., Lazar, M., & Field, A. S. (2007). Diffusion tensor imaging of the brain. *Neurotherapeutics : The Journal of the American Society for Experimental NeuroTherapeutics*, *4*(3), 316–329. https://doi.org/10.1016/j.nurt.2007.05.011

Andersson, J. L. R., Jenkinson, M., & Smith, S. (2007a). Non-linear registration, aka spatial normalisation. FMRIB Technial Report TR07JA2. *Oxford Centre for Functional Magnetic Resonance Imaging of the Brain, Department of Clinical Neurology, Oxford University, Oxford, UK*, (June), 22. https://doi.org/10.1016/j.neuroimage.2008.10.055

Andersson, J. L. R., Jenkinson, M., & Smith, S. M. (2007b). Non-linear optimisation. FMRIB technical report TR07JA1. *In Practice*, (June), 16. Retrieved from http://fsl.fmrib.ox.ac.uk/analysis/techrep/tr07ja1/tr07ja1.pdf

Andersson, J. L. R., Skare, S., & Ashburner, J. (2003). How to correct susceptibility distortions in spin-echo echo-planar images: Application to diffusion tensor imaging. *NeuroImage*, *20*(2), 870–888. https://doi.org/10.1016/S1053-8119(03)00336-7

Andersson, J. L. R., & Sotiropoulos, S. N. (2016). An integrated approach to correction for off-resonance effects and subject movement in diffusion MR imaging. *NeuroImage*, *125*, 1063–1078. https://doi.org/10.1016/j.neuroimage.2015.10.019

Basser, P. J., Mattiello, J., & LeBihan, D. (1994). MR diffusion tensor spectroscopy and imaging. *Biophysical Journal*, *66*(1), 259–267. https://doi.org/10.1016/S0006-3495(94)80775-1

Dhollander, T., Raffelt, D., & Connelly, A. (2016). Unsupervised 3-tissue response function estimation from single-shell or multi-shell diffusion MR data without a co-registered T1 image. *ISMRM Workshop on Breaking the Barriers of Diffusion MRI*, (September), 5. Retrieved from https://www.researchgate.net/publication/307863133_Unsupervised_3-tissue_response_function_estimation_from_single-shell_or_multi-shell_diffusion_MR_data_without_a_co-registered_T1_image

Hua, K., Zhang, J., Wakana, S., Jiang, H., Li, X., Reich, D. S., … Mori, S. (2008). Tract Probability Maps in Stereotaxic Spaces: Analyses of White Matter Anatomy and Tract-Specific Quantification. *Neuroimage*, *39*(1), 336–347. https://doi.org/10.1055/s-0029-1237430.Imprinting

Jenkinson, M., Beckmann, C. F., Behrens, T. E. J., Woolrich, M. W., & Smith, S. M. (2012, August 15). FSL. *NeuroImage*, Vol. 62, pp. 782–790. https://doi.org/10.1016/j.neuroimage.2011.09.015

Mori, S., & Zijl, P. Van. (2007). Human white matter atlas. *American Journal of Psychiatry*, *164*(July), 75390. https://doi.org/10.1176/appi.ajp.164.7.1005

Pasternak, O., Sochen, N., Gur, Y., Intrator, N., & Assaf, Y. (2009). Free water elimination and mapping from diffusion MRI. *Magnetic Resonance in Medicine*, *62*(3), 717–730. https://doi.org/10.1002/mrm.22055

Roalf, D. R., Quarmley, M., Elliott, M. A., Satterthwaite, T. D., Vandekar, S. N., Ruparel, K., … Gur, R. E. (2016). The Impact of Quality Assurance Assessment on Diffusion Tensor Imaging Outcomes in a Large-Scale Population-Based Cohort. *NeuroImage*, *125*, 903–919. https://doi.org/10.1016/j.neuroimage.2015.10.068.

Skare, S., & Andersson, J. L. (2001). On the effects of gating in diffusion imaging of the brain using single shot EPI. *Magnetic Resonance Imaging*, *19*(8), 1125–1128. https://doi.org/10.1016/S0730-725X(01)00415-5

Smith, S. M., Jenkinson, M., Johansen-Berg, H., Rueckert, D., Nichols, T. E., Mackay, C. E., … Behrens, T. E. J. (2006). Tract-based spatial statistics: Voxelwise analysis of multi-subject diffusion data. *NeuroImage*, *31*(4), 1487–1505. https://doi.org/10.1016/j.neuroimage.2006.02.024

Smith, S. M., Jenkinson, M., Woolrich, M. W., Beckmann, C. F., Behrens, T. E. J., Johansen-berg, H., … Matthews, P. M. (2004). Advances in Functional and Structural MR Image Analysis and Implementation as FSL Technical Report TR04SS2. *Neuroimage*, *23(S1)*, 208–219. https://doi.org/10.1016/j.neuroimage.2004.07.051

Song, S. K., Sun, S. W., Ju, W. K., Lin, S. J., Cross, A. H., & Neufeld, A. H. (2003). Diffusion tensor imaging detects and differentiates axon and myelin degeneration in mouse optic nerve after retinal ischemia. *NeuroImage*, *20*(3), 1714–1722. https://doi.org/10.1016/j.neuroimage.2003.07.005

Song, S. K., Sun, S. W., Ramsbottom, M. J., Chang, C., Russell, J., & Cross, A. H. (2002). Dysmyelination revealed through MRI as increased radial (but unchanged axial) diffusion of water. *NeuroImage*, *17*(3), 1429–1436. https://doi.org/10.1006/nimg.2002.1267

Veraart, J., Fieremans, E., & Novikov, D. S. (2016). Diffusion MRI noise mapping using random matrix theory. *Magnetic Resonance in Medicine*, *76*(5), 1–12. https://doi.org/10.1002/mrm.26059.

Veraart, J., Novikov, D. S., Christiaens, D., Ades-aron, B., Sijbers, J., & Fieremans, E. (2016). Denoising of diffusion MRI using random matrix theory. *NeuroImage*, *142*(November 15), 394–406. https://doi.org/10.1016/j.neuroimage.2016.08.016.

Veraart, J., Sijbers, J., Sunaert, S., Leemans, A., & Jeurissen, B. (2013). Weighted linear least squares estimation of diffusion MRI parameters: Strengths, limitations, and pitfalls. *NeuroImage*, *81*, 335–346. https://doi.org/10.1016/j.neuroimage.2013.05.028

Zhang, Y., Brady, M., & Smith, S. (2001). Segmentation of brain MR images through a hidden Markov random field model and the expectation-maximization algorithm. *IEEE Trans.Med.Imaging*, *20*(0278–0062), 45–57. https://doi.org/10.1109/42.906424
